# Histone deacetylase 3 promotes liver regeneration and liver cancer cells proliferation through signal transducer and activator of transcription 3 signaling pathway

**DOI:** 10.1038/s41419-018-0428-x

**Published:** 2018-03-14

**Authors:** Xu-Feng Lu, Xiao-Yue Cao, Yong-Jie Zhu, Zhen-Ru Wu, Xiang Zhuang, Ming-Yang Shao, Qing Xu, Yong-Jie Zhou, Hong-Jie Ji, Qing-Richard Lu, Yu-Jun Shi, Yong Zeng, Hong Bu

**Affiliations:** 1 Laboratory of Pathology, Key Laboratory of Transplant Engineering and Immunology, NHFPC; West China Hospital, Sichuan University, Chengdu, 610041 China; 2 Department of Pathology, West China Hospital, Sichuan University, Chengdu, 610041 China; 3 Department of Pediatrics, Division of Experimental Hematology and Cancer Biology, Brain Tumor Center, Cincinnati Children’s Hospital Medical Center, Cincinnati, OH 25229 USA; 4 Department of Liver and Vascular Surgery, West China Hospital, Sichuan University, Chengdu, 610041 China

## Abstract

Histone deacetylase 3 (HDAC3) plays pivotal roles in cell cycle regulation and is often aberrantly expressed in various cancers including hepatocellular carcinoma (HCC), but little is known about its role in liver regeneration and liver cancer cells proliferation. Using an inducible hepatocyte-selective HDAC3 knockout mouse, we find that lack of HDAC3 dramatically impaired liver regeneration and blocked hepatocyte proliferation in the G1 phase entry. HDAC3 inactivation robustly disrupted the signal transducer and activator of transcription 3 (STAT3) cascade. HDAC3 silencing impaired the ac-STAT3-to-p-STAT3 transition in the cytoplasm, leading to the subsequent breakdown of STAT3 signaling. Furthermore, overexpressed HDAC3 was further associated with increased tumor growth and a poor prognosis in HCC patients. Inhibition of HDAC3 expression reduced liver cancer cells growth and inhibited xenograft tumor growth. Our results suggest that HDAC3 is an important regulator of STAT3-dependent cell proliferation in liver regeneration and cancer. These findings provide novel insights into the HDAC3–STAT3 pathway in liver pathophysiological processes.

## Introduction

The mammalian liver has an enormous regenerative ability to restore its original mass and function via compensatory hyperplasia in response to diverse injuries^1^. Quiescent maturity hepatocytes in the G0 phase of the cell cycle can re-enter into the cell cycle rapidly to produce new hepatocytes following partial hepatectomy (PH) or chemical injury^2^. In humans, liver regeneration proceeds within weeks of a major liver resection, whereas in rodents, liver recovery can occur within 10 days of PH^1^. Aberrant liver regeneration is closely related to the pathogenesis of liver failure or hepatocellular carcinoma (HCC)^3^. A better understanding of the basic mechanisms that regulate liver regeneration will have important implications for potential therapies for human liver disease.

Histone deacetylase 3 (HDAC3), a member of the highly conserved Class I HDAC enzymes, is ubiquitously expressed in eukaryotic cells^4^. HDAC3 regulates gene transcription by catalyzing the deacetylation of core histones and is involved in various biological processes in the liver^5^. Inhibition of HDAC3 and other HDACs specifically interferes with liver development in zebrafish^6^. In mammals, hepatic-specific ablation of HDAC3 dramatically disrupts liver function and causes severe dysregulation of lipid and glucose metabolism^7,8^. Conversely, high HDAC3 expression in the liver was found to contribute to high-fat-diet-induced metabolic syndrome^9^. HDAC3 is also essential for the maintenance of chromatin structure and genomic stability, and hepatic loss of HDAC3 in mice results in an accumulation of DNA damage and the early onset of spontaneous liver cancer^10,11^. Still, the role of HDAC3 in HCC development remains unclear because the mRNA and protein levels of HDAC3 can either increase or decrease in human HCC tissues^11,12^. Furthermore, HDAC3 is a considered biomarker of tumor recurrence following liver transplantation in hepatitis B virus-associated HCC^13^. HDAC3 also participates in the self-renewal process of liver cancer stem cells through histone modifications^14^, making HDAC3 suppression a potential clinical-therapeutic approach for HCC.

Multiple *in vitro* studies have revealed that HDAC3 plays diverse roles in the regulation of cell cycle progression. HDAC3 is required to generate a hypoacetylated H3 tail as the preferred template for the phosphorylation of H3S10 by Aurora B, which is a crucial step for progression through the G2/M phase and mitosis^15^. HDAC3 is also required for H3K4 deacetylation at the centromere and sister chromatin cohesion^16,17^. HDAC3 inhibition induces G1 phase arrest by increasing p21^WAF1/cip1^ expression in Hep3B liver cancer cells^18^. HDAC3 deletion in mouse embryonic fibroblasts induces S-phase arrest through DNA damage accumulation, whereas inactivation of HDAC3 affects replication fork progression in hematopoietic progenitor cells during the S phase^10,19^. HDAC3 also controls G2/M progression in adult neural stem/progenitor cells by regulating the CDK1 levels^20^. In addition, HDAC3 regulates cell cycle progression by modulating cyclin A acetylation to control cyclin A level^21^. The data regarding the role and molecular mechanism of HDAC3 in regulating cell proliferation are highly multiple and contradictory. However, the biological function of HDAC3 in liver regeneration and liver cancer cells proliferation remains unknown.

In this study, we determined that HDAC3 promotes liver regeneration and liver cancer cells proliferation through the signal transducer and activator of transcription 3 (STAT3) signaling pathway. The role of HDAC3 in the regulation of cell proliferation through the STAT3 pathway provides a potential drug target for the treatment of HCC.

## Results

### HDAC3 deletion severely impairs liver regeneration by delaying the G1 phase entry

**Fig. 1:**
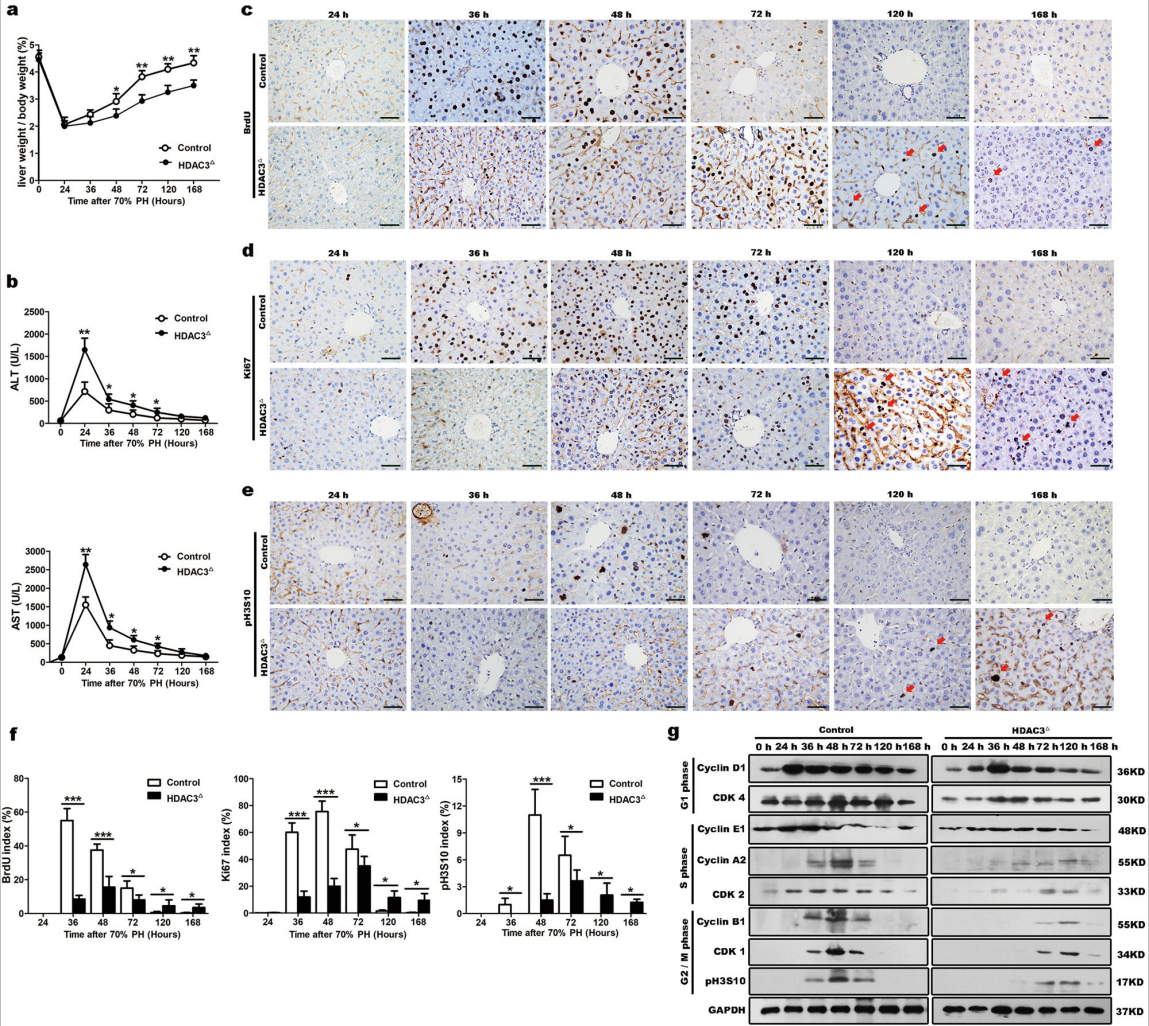
Impaired liver regeneration in HDAC3^△^ mice following 70% PH. **a** The liver weight/body weight ratio was calculated at different time points after PH, and liver reconstitution was notably delayed in the HDAC3^△^ livers. **b** The serum AST and ALT levels after PH. **c**–**f** Immunohistochemistry for BrdU, Ki67, and p-Histone H3 (Ser10) shows that the proliferation rates are reduced in the HDAC3^△^ mice after PH (scale bar: 50 μm). **g** Western blot analysis of cell cycle proteins at different times after PH shows that mitotic hepatocytes are delayed at the G1 phase entrance in the HDAC3^△^ mice. The levels of the indicated proteins are expressed relative to GAPDH. All data represent the mean ± SD; *n* = 3–8; *p < 0.05; **p < 0.01; ***p < 0.001

As described previously^7,10^, in the Alb-Cre;HDAC3^loxP/loxP^ mouse, where HDAC3 is constitutively inactivated, dramatic dysregulation of hepatic metabolism and notable damage to the DNA are evident at 2 months of age (Supplementary Fig. 1). Therefore, to eliminate post-natal developmental defects upon HDAC3 deletion, we generated an inducible tamoxifen-dependent hepatocyte-selective HDAC3 knockout mouse (Alb-Cre^ERT2^;HDAC3^loxP/loxP^ mouse), in which HDAC3 in the liver tissues was over 80% ablated after 5 days of tamoxifen treatment (hereafter referred to as the HDAC3^△^ mouse) (Supplementary Fig. 2). Five days after the last injection of tamoxifen, the inducible HDAC3^△^ mouse did not show obviously impaired liver function or disrupted DNA stability (Supplementary Fig. 2). Next, we performed two-thirds PH of the livers in male HDAC3^△^ mice and their control littermates (Alb-Cre^ERT2^;HDAC3^loxP/loxP^ mice treated with corn oil instead of tamoxifen) to explore the specific role of HDAC3 during liver regeneration. Surprisingly, the post-hepatectomy mutant mice displayed a severely impaired liver mass reconstitution, which was calculated using the liver weight/body weight ratio (Fig. 1a). Furthermore, HDAC3 silencing interfered with liver recovery as revealed by the alanine transaminase and aspartate transaminase serum levels (Fig. 1b). The bromodeoxyuridine (BrdU) incorporation and Ki67 immunohistochemistry showed that the proliferation peak in the control littermates appeared between 36 and 48 h post-hepatectomy; strikingly, HDAC3 disruption reduced the proliferation rates by approximately 60% during this time (Fig. 1c, d, f). Phospho-histone H3S10 (p-H3S10), a mitotic marker that is specifically expressed during the G2/M phase^15^, showed a corresponding decrease that was over three-fold in the HDAC3^△^ mice at 48 h post-hepatectomy (Fig. 1e, f). The livers of the control mice were completely reconstituted within 168 h, and mitotic hepatocytes were undetectable by 168 h; however, frequent proliferation was observed in the HDAC3^△^ mice until 14 days after the PH (Supplementary Fig. 3a).

Similarly, when the mice were challenged with a carbon tetrachloride (CCl_4_) injection, the absence of HDAC3 did not increase sensitivity to the toxin but robustly delayed liver repair (Supplementary Fig. 4).

We next examined potential differences in the expression levels of key cell cycle markers between the HDAC3^△^ and control mice during liver regeneration. Ki67 is expressed from mid-G1 to the end of mitosis^22^, and the low Ki67 staining indicates that the proliferating hepatocytes might be arrested in the early G1 phase. Consistently, the levels of cyclin D1 and CDK4, which are the key markers of the early G1 phase^22^, peaked at 24 h after PH in the control livers; however, the cyclin D1 and CDK4 levels were dramatically suppressed in the HDAC3^△^ livers (Fig. 1g; Supplementary Fig. 3b). The levels of CDK2 and cyclin E1, which function at the G1/S transition^22^, peaked at 36 h post-hepatectomy, but the levels were correspondingly lower in the mutant livers (Fig. 1g; Supplementary Fig. 3b). Notably, the protein levels of cyclin A2, an essential marker for S-phase progression^22^, were significantly reduced in the HDAC3^△^ livers at 48 h after PH (Fig. 1g; Supplementary Fig. 3b). Additionally, the levels of CDK1, cyclin B1, and p-H3S10, which appear in the G2/M phase^22^, were also markedly decreased in the HDAC3^△^ livers during the peak period of mitosis (Fig. 1g; Supplementary Fig. 3b). Taken together, our findings suggested that HDAC3-deficient hepatocytes were robustly arrested in the G1 phase entry.

### HDAC3 inactivation impairs STAT3 signaling

**Fig. 2:**
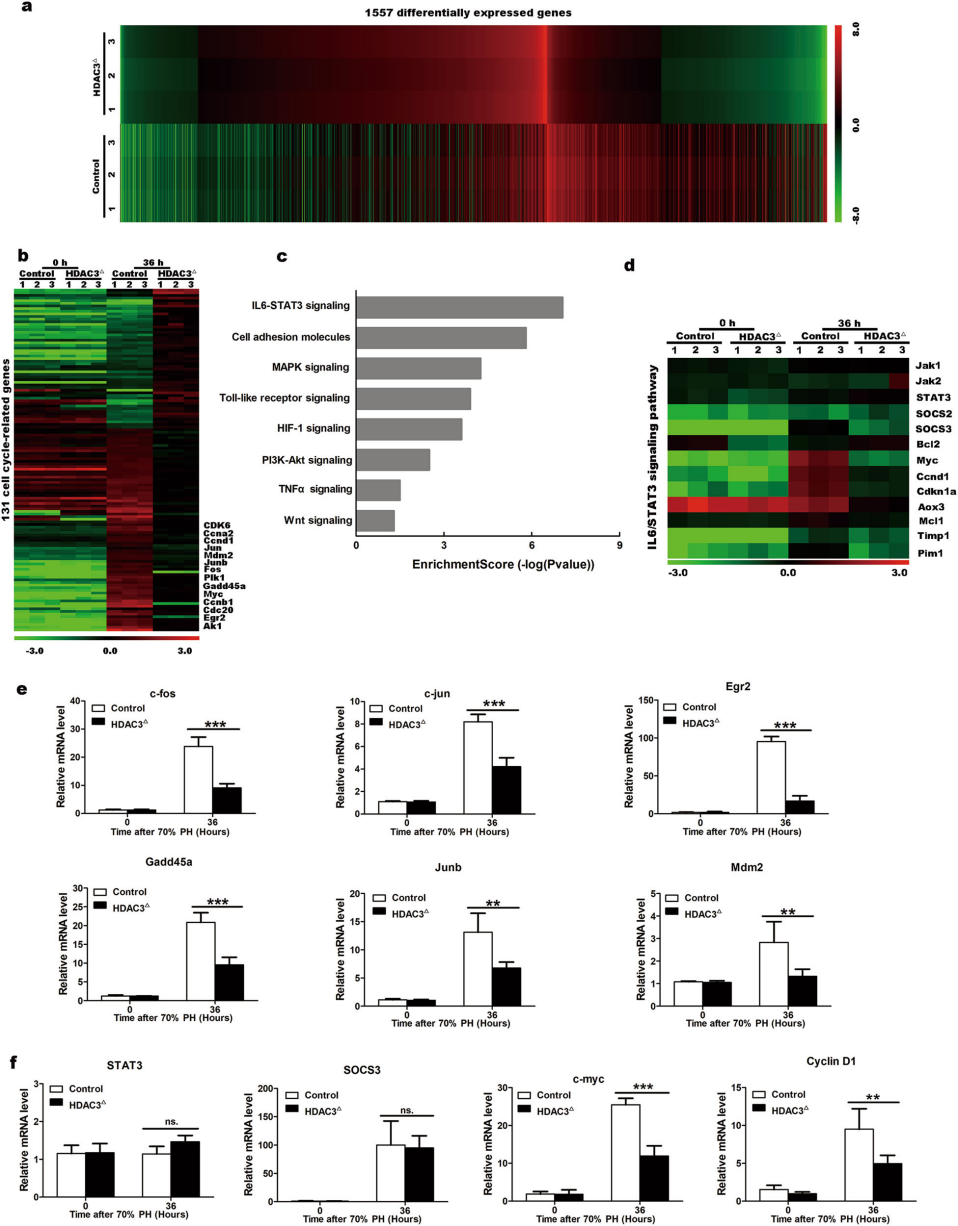
The gene expression profile demonstrates the broad and multiple effects of HDAC3 on gene expression after PH. **a** A heat map of 1557 differentially expressed genes (fold changes ≥1.5 and *p* < 0.05) in the control and mutant livers at 36 h after PH compared with their corresponding baseline values (0 h). **b** mRNA microarray analysis of cell cycle-related genes. Differentially expressed genes (fold changes ≥1.25 and *p* < 0.05) in the control and HDAC3^△^ livers at 36 h after PH. **c** GO term analysis of the list of differentially expressed genes (fold changes ≥1.5 and *p* < 0.05) indicated that the IL6/STAT3 pathway is dramatically changed during liver regeneration in the HDAC3^△^ mice after PH. **d** Heat map of the selected genes from the microarray analysis of the control and HDAC3^△^ livers. **e** Quantitative RT-PCR confirmation of the downregulated early-response genes in the HDAC3^△^ livers vs. the control liver at 36 h after PH. **f** qRT-PCR confirms downregulation of cyclin D1 and c-myc in the HDAC3^△^ livers vs. the control liver at 36 h after PH. All data represent the mean ± SD; *n* = 3; **p < 0.01; ***p < 0.001; n.s. not significant

Because the rapid transitions of the cell cycle activate multiple signaling cascades and numerous genes during liver regeneration^1^, we performed a microarray analysis to screen the molecular defects in the HDAC3^△^ liver. The inducible short-term absence of HDAC3 did not obviously alter the gene expression profiles of the quiescent hepatocytes (Supplementary Fig. 5a–c); however, the gene profiles of the mutant liver were dramatically changed 36 h after the PH (Fig. 2a). Among these genes, 131 cell cycle-related genes were identified, and cyclin D1, cyclin A2, cyclin B1, and CDK1 were significantly downregulated in the HDAC3^△^ liver (Fig. 2b; Supplementary Fig. 5d), which was consistent with the proliferation defects observed in the mutant liver.

A Gene Ontology (GO) term analysis of the differentially regulated genes showed that the interleukin 6 (IL6)/STAT3 signaling pathway, which regulates approximately 36% of the immediate-early genes during the acute hepatic response^1^, was extremely disturbed (Fig. 2c, d). As expected, the immediate early-response genes, including c-fos, c-jun, Egr2/3, Gadd45a, junb, and Mdm2^23^, were significantly downregulated in the HDAC3^△^ livers (Fig. 2e, Supplementary Fig. 5d). Additionally, c-myc and cyclin D1, which are components of the STAT3 pathway^24^, were among the most reduced genes, and this observation was further confirmed by qRT-PCR (Fig. 2f). Collectively, our microarray data supported the idea that breakdown of STAT3 signaling in the HDAC3^△^ mice might account for the G1 phase arrest during liver regeneration.

### HDAC3 silencing impairs the STAT3 transition from acetylation to phosphorylation

**Fig. 3:**
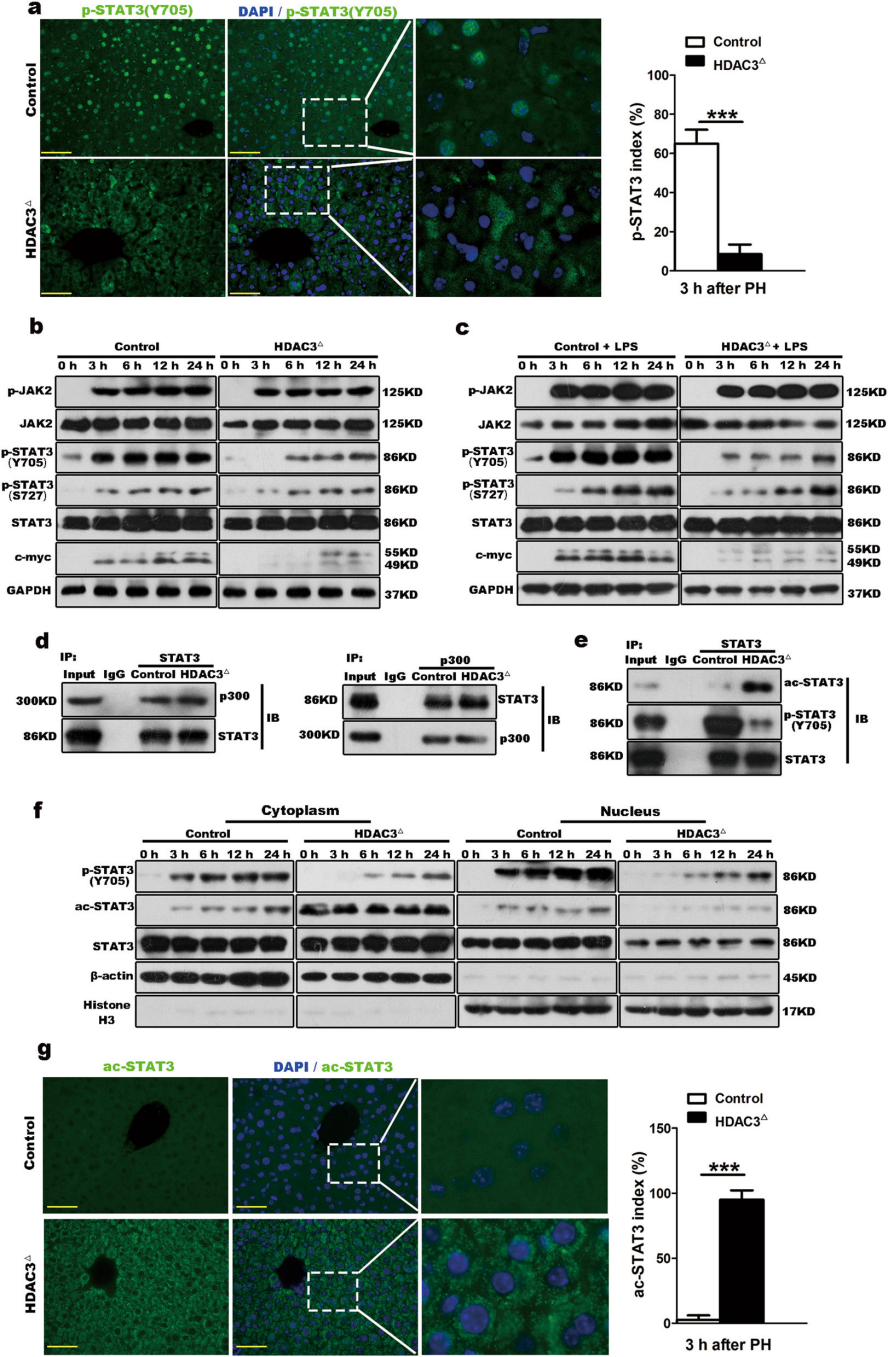
The HDAC3 deficiency inhibited STAT3(Y705) phosphorylation and enhanced STAT3 acetylation in hepatocytes. **a** Immunofluorescence demonstrates that p-STAT3(Y705) is not activated in the HDAC3^△^ hepatocytes (scale bar: 50 μm). **b** Western blot analysis shows that p-STAT3(Y705) and c-myc are reduced in the HDAC3^△^ mice during the early phase of liver regeneration. GAPDH was used as the loading control. **c** Western blot analysis shows that p-STAT3 remains inhibited in the mutant hepatocytes after the LPS/PH treatment. **d** Co-immunoprecipitation shows that STAT3 is associated with p300 in both the control and HDAC3^△^ liver. **e** Immunoprecipitation confirms that the protein complex overwhelmingly consists of ac-STAT3 with extremely low p-STAT3(Y705) in the HDAC3^△^ liver. **f** Western blot analysis shows that the HDAC3 deficiency leads to prolonged acetylation of STAT3 in the cytoplasm during liver regeneration. β-Actin and histone H3 were used as the loading controls. **g** Immunofluorescence demonstrates that ac-STAT3 significantly accumulates in the cytoplasm of the HDAC3^△^ hepatocytes (scale bar: 50 μm). *n* = 3–5; ***p < 0.001

STAT3 mediates the expression of various genes in response to diverse stimuli and thus plays a key role in multiple cellular processes such as cell growth, proliferation, and apoptosis^24^. When IL6 combines with its membrane receptor glycoprotein 130 (gp130), STAT3 becomes phosphorylated at tyrosine^705^ (Y705) by receptor-associated Janus kinases (JAKs)^24^. Compared with the control littermates, in which a large number of p-STAT3(Y705) nuclear-positive hepatocytes were observed at 3 h after PH, nuclear p-STAT3(Y705) was abolished in HDAC3-deficient mice (Fig. 3a–c; Supplementary Fig. 6a). Additionally, the level of c-myc, which is a major downstream gene of STAT3 signaling and an important transcription factor for successful initiation of cell mitosis^23^, was also reduced in HDAC3-deficient mice at the early phase post-hepatectomy (Fig. 3b; Supplementary Fig. 6a). Intriguingly, the level of p-STAT3(S727), which is phosphorylated by the c-src non-receptor tyrosine kinase^25^, was not obviously impaired in the HDAC3-deficient livers (Fig. 3b), indicating that HDAC3 ablation might selectively target p-STAT3(Y705). To further determine whether the low level of p-STAT3(Y705) was caused by the HDAC3 deletion, we examined p-STAT3(Y705) expression in HDAC3-deficient mice by simultaneously challenging the mice with PH and intraperitoneal injections of lipopolysaccharide (LPS) (Supplementary Fig. 6b, c). IL6 is extraordinarily increased by LPS stimulation^26^, whereas the levels of p-STAT3(Y705) and c-myc remained notably depressed in the HDAC3^△^ livers, regardless of the upregulation of p-JAK2 (Fig. 3c; Supplementary Fig. 6c).

Upon ligand stimulation, acetylation of STAT3 (ac-STAT3) is critical for enhancing the transcription of cell growth-related genes^27,28^. Co-activator p300, which is a histone acetyltransferase that primarily functions in the nucleus, is required for STAT3 acetylation^29,30^. Our immunoprecipitation assay showed a strong interaction between p300 and STAT3 in both the control and HDAC3^△^ livers (Fig. 3d). As STAT3 activity is modified by phosphorylation and acetylation at post-translational level^27^, we next examined whether HDAC3 deficiency results in impaired STAT3 activation. In the mutant liver, the protein that was co-immunoprecipitated by the STAT3 antibody overwhelmingly consisted of ac-STAT3 with extremely low p-STAT3(Y705), while the control liver showed the opposite dynamic (Fig. 3e). The absence of p-STAT3(Y705) in the nucleus prompted us to investigate whether HDAC3 deprivation impaired the STAT3 transition from acetylation to phosphorylation during liver regeneration. The cytoplasmic and nuclear fractions were prepared from the livers after PH at indicated time points, and the immunoblotting analysis showed that during the early phase of liver regeneration, p-STAT3(Y705) was notably increased both in the cytoplasm and nucleus of the control hepatocytes whereas ac-STAT3 was maintained at a low level in the cytoplasm (Fig. 3f). Conversely, HDAC3-deficient hepatocytes showed substantial hyperacetylation of STAT3 but notably reduced p-STAT3(Y705) in both the cytoplasm and the nucleus (Fig. 3f, g).

### HDAC3 deletion impairs cell growth by inhibiting nuclear translocation of STAT3

**Fig. 4:**
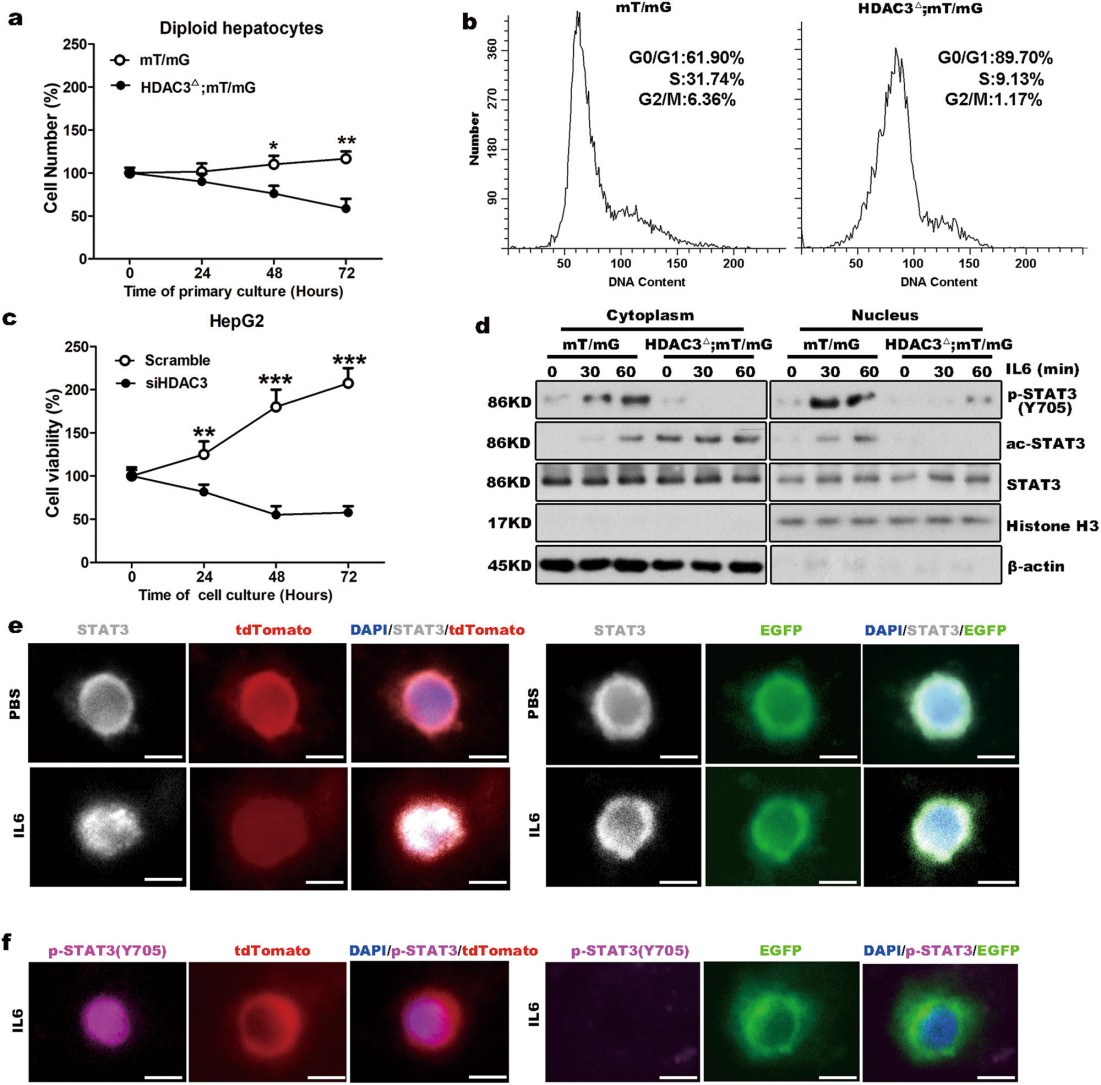
The HDAC3 deficiency suppresses hepatocyte proliferation and STAT3 signaling *in vitro*. **a** Living hepatocyte numbers show that cell number of the mutant diploid hepatocyte is continuously decreased. The values are normalized to the average cell numbers on 0 h. **b** The cell cycle of *in vitro* diploid hepatocytes following mitogen stimulation for 48 h was determined by flow cytometry analysis. **c** Cell growth in HepG2 cells was tested after HDAC3 knockdown using a CCK-8 kit. **d** Western blot analysis demonstrates that STAT3(Y705) fails to be phosphorylated due to the high level of ac-STAT3 in the primary mutant hepatocytes after the IL6 treatment. β-Actin and histone H3 were used as the loading controls. **e** Immunofluorescence shows that STAT3 is unable to accumulate in the nuclei of the mutant hepatocytes following IL6 stimulation for 30 min (scale bar: 5 μm). **f** Immunofluorescence demonstrates that p-STAT3(Y705) remains absent in the mutant hepatocytes after the IL6 treatment (scale bar: 5 μm). All data represent the mean ± SD; *n* = 3–5; *p < 0.05; **p < 0.01; ***p < 0.001

To determine whether the HDAC3 deficiency inhibited the nuclear translocation of STAT3, we cultured fluorescently labeled primary diploid hepatocytes that were isolated from the (Gt(ROSA)26Sortm4(ACTB-tdTomato-,EGFP) mice (hereafter referred to as mT/mG mice) and HDAC3^△^;mT/mG mice, respectively (Supplementary Fig. 7). Cell viability assays revealed that compared to the diploid hepatocytes that were isolated from the mT/mG mice, cell number of the HDAC3 mutant hepatocytes was significantly decreased (Fig. 4a, b). Consistently, HDAC3 knockdown in human liver cancer cells HepG2 using siRNA significantly increased ac-STAT3 expression level and remarkably inhibited cell growth (Fig. 4c; Supplementary Fig. 8a–c).

Notably, the mutant hepatocytes displayed robustly decreased p-STAT3(Y705) with significantly increased cytoplasmic ac-STAT3; additionally, IL6 stimulation failed to upregulate p-STAT3(Y705) in the HDAC3-deficient cells (Fig. 4d). Because STAT3 localizes to the nucleus after it is phosphorylated^24^, we tested whether ac-STAT3 prevented the nuclear translocation of STAT3. Indeed, the immunofluorescence analysis showed that the nuclear localization of total STAT3 was significantly suppressed in the HDAC3-deficient hepatocytes after IL6 treatment (Fig. 4e). Moreover, p-STAT3(Y705) was noticeably localized to the nuclei of the control hepatocytes, whereas p-STAT3(Y705) remained absent from the HDAC3-deficient hepatocyte nuclei, even with IL6 stimulation (Fig. 4f). Meanwhile, the phosphorylation of STAT3(Y705) after IL6 stimulation was also prevented in response to HDAC3 knockdown (Supplementary Fig. 8b–d), which indicated that loss of HDAC3 remarkably disrupted the transduction of STAT3 signaling from the cytoplasm to the nucleus.

### HDAC3 is essential for deacetylation of ac-STAT3 in the cytoplasm

**Fig. 5:**
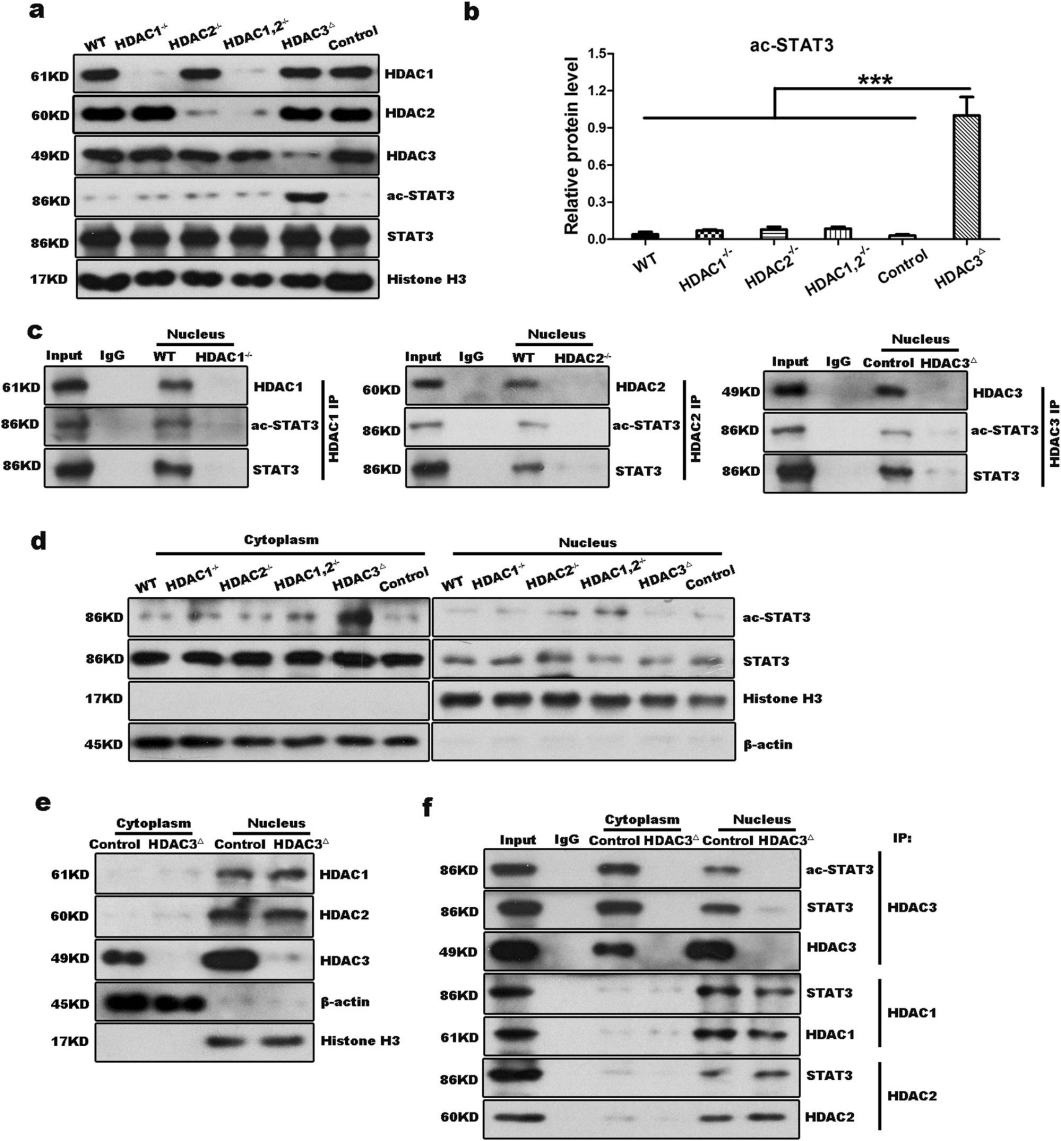
HDAC3 specifically interacts with STAT3 in the cytoplasm. **a**,** b** Western blot analysis demonstrates that the level of ac-STAT3 is high in the HDAC3^△^ liver at 6 h after PH. Histone H3 was used as the loading control. **c** Immunoprecipitation shows that HDAC1, HDAC2, and HDAC3 can each combine with ac-STAT3 in the nucleus following PH. **d** Immunoblotting to determination of the cytoplasmic and nuclear distribution shows that ac-STAT3 is primarily localized to the cytoplasm at 6 h after PH. β-Actin and histone H3 were used as the loading controls. **e** Immunoblotting to determination of the cytoplasmic and nuclear distributions of HDAC1, HDAC2, and HDAC3 in the control and HDAC3^△^ mice demonstrates that HDAC3 localizes to both the cytoplasm and nucleus, whereas HDAC1 and HDAC2 strictly localize to the nucleus. **f** Co-immunoprecipitation assays demonstrate that HDAC3, but not HDAC1 or HDAC2, combines with STAT3 in the cytoplasm of hepatocytes from control livers. All data represent the mean ± SD; *n* = 3–5; ***p < 0.001

Class I HDACs have been suggested to contribute to STAT3 deacetylation in 293T cells^29^, but the individual roles of the Class I HDAC members in the deacetylation of ac-STAT3 *in vivo* remain undefined. We previously reported that loss of HDAC1 and/or HDAC2 did not disturb cell cycle progression before the M phase^31^, which highlighted the negative effects of HDAC1 and HDAC2 on the IL6/STAT3 signaling cascade. Consistently, deletions of HDAC1, HDAC2, or both did not increase the level of ac-STAT3 compared to that of the wild-type (WT) liver during the early phase of liver regeneration (Fig. 5a, b). Moreover, loss of HDAC8, another member of the Class I HDACs, did not have a noticeable effect on the progression of liver regeneration (Supplementary Fig. 9). Therefore, we did not further analyze the role of HDAC8 in STAT3 signaling during liver regeneration.

It has been suggested that ac-STAT3 is likely to be deacetylated by Class I HDACs in the nucleus^28,32^. To test this, we performed immunoprecipitations to examine the associations between STAT3 and HDAC1, HDAC2, and HDAC3 in the nucleus. All three Class I HDAC members interacted with ac-STAT3 (Fig. 5c); however, compared to the WT liver, single disruptions to each HDAC or a combined disruption of HDAC1 and HDAC2 did not noticeably increase the nuclear level of ac-STAT3 following PH (Fig. 5d). This observation strongly suggested that HDACs do not catalyze the deacetylation of ac-STAT3 in the nucleus. This observation was also validated by the finding that high levels of ac-STAT3 were evident in the cytoplasm of hepatocytes from each genotypic mouse, though the ac-STAT3 levels were much higher in the HDAC3-deficient livers than in the HDAC1- and/or HDAC2-deficient livers (Figs. 3g and 5d).

The cytoplasmic but not nuclear accumulation of ac-STAT3 in the HDAC3-deficient liver strongly indicated that ac-STAT3 is primarily deacetylated in the cytoplasm rather than in the nucleus, and HDAC3 might function a powerful role in the deacetylation of ac-STAT3. Although HDAC3 is a nuclear protein, HDAC3 contains a nuclear export signal in its C terminus (residues 180–313) which facilitates the nucleoplasm transport of HDAC3^33,34^. We confirmed the cytoplasmic distribution of HDAC3 by immunoblotting, and in contrast to HDAC3, HDAC1 and HDAC2 were nearly absent from the hepatocyte cytoplasm (Fig. 5e). Moreover, HDAC3, but not HDAC1 or HDAC2, was associated with ac-STAT3 in the cytoplasm (Fig. 5f; Supplementary Fig. 8e), suggesting that HDAC3 played a unique role in the deacetylation of ac-STAT3 in the cytoplasm.

### HDAC3 enhances p-STAT3(Y705) and Ki67 index in HDAC3-positive HCC

**Fig. 6:**
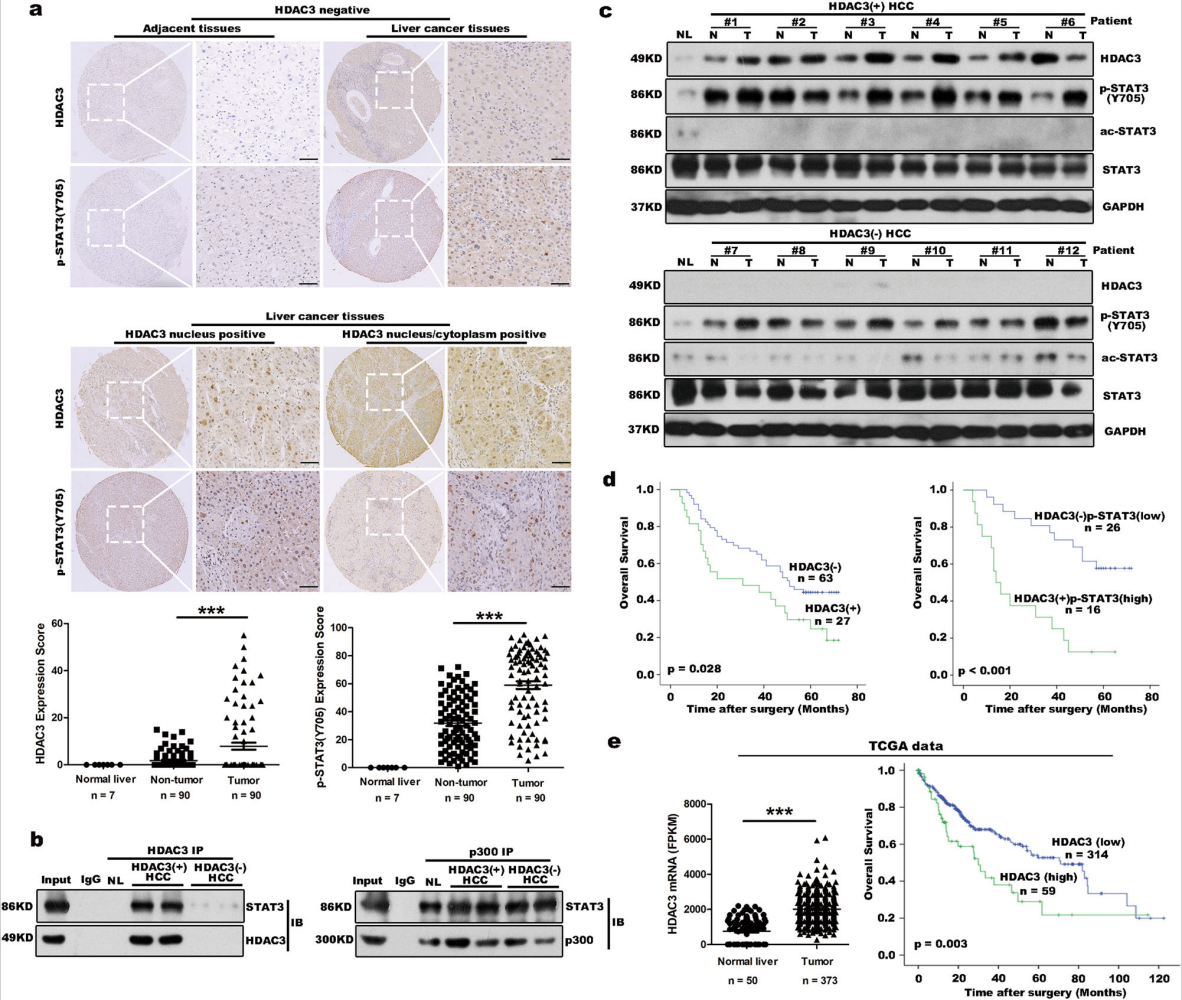
HDAC3 is highly expressed in HCC subtypes and is correlated with a poor prognosis in HCC patients. **a** Representative microphotograph and statistical analysis of HDAC3 and p-STAT3(Y705) expression in HCC tissues (*n* = 90) by immunohistochemistry (scale bar: 50 μm). **b** Co-immunoprecipitation shows the combination between STAT3 and HDAC3 or p300 in HDAC3-positive (+) or HDAC3-negative (−) HCC tissues. **c** Immunoblotting demonstrates that p-STAT3(Y705) increases in HDAC3-positive HCC tissues. NL normal liver, T tumor tissue, NT adjacent non-tumor tissues. GAPDH was used as the loading control. **d** Kaplan–Meier analysis shows that single upregulation of HDAC3 (*n* = 90) or combined upregulation of HDAC3 and p-STAT3(Y705) (*n* = 42) significantly reduces the overall survival rate of HCC patients. **e** Analysis of TCGA data revealed that HCC patients with high HDAC3 expression (*Z* score >1) were significantly associated with a poorer overall survival. FPKM fragments per kilobase of exon per million fragments mapped. All data represent the mean ± SD; ***p < 0.001

Active proliferation of tumor cells is a major feature of HCC that often indicates a poor prognosis^35^. The IL6/STAT3 signaling pathway plays crucial roles in the tumorigenesis and development of HCC^36^. Because HDAC3 is a critical regulator of STAT3 signaling during liver regeneration, we investigated whether elevated HDAC3 in HCC was correlated with a higher tumor cell proliferation rate. We performed immunohistochemistry to assess HDAC3 expression in tissues from 90 HCC cases, and observed that 27 cases exhibited strong nuclear and cytoplasmic HDAC3 positivity, and that HDAC3 was either negatively or weakly expressed in the corresponding adjacent non-tumor tissues (Fig. 6a; Supplementary Table 4). As observed by immunoprecipitation, HDAC3 was also detected in combination with STAT3 in HDAC3-positive HCC (Fig. 6b), highlighting their interaction. Remarkably, HDAC3 was positively correlated with p-STAT3 and the Ki67 index in the HDAC3-positive HCCs (Pearson correction = 0.622, *P* < 0.001, and Pearson correction = 0.760, *P* < 0.001, respectively) (Fig. 6c; Supplementary Fig. 10). The Kaplan–Meier analysis showed that the high expression of HDAC3 alone or in combination with high p-STAT3(Y705) remarkably reduced recurrence-free survival (Fig. 6d; Supplementary Tables 5 and 6). Consistently, the Cancer Genome Atlas (TCGA) data also revealed that HCC patients with high HDAC3 expression have lower overall survival rates (Fig. 6e).

### Inhibition of HDAC3 expression decreases HCC xenografts growth

**Fig. 7:**
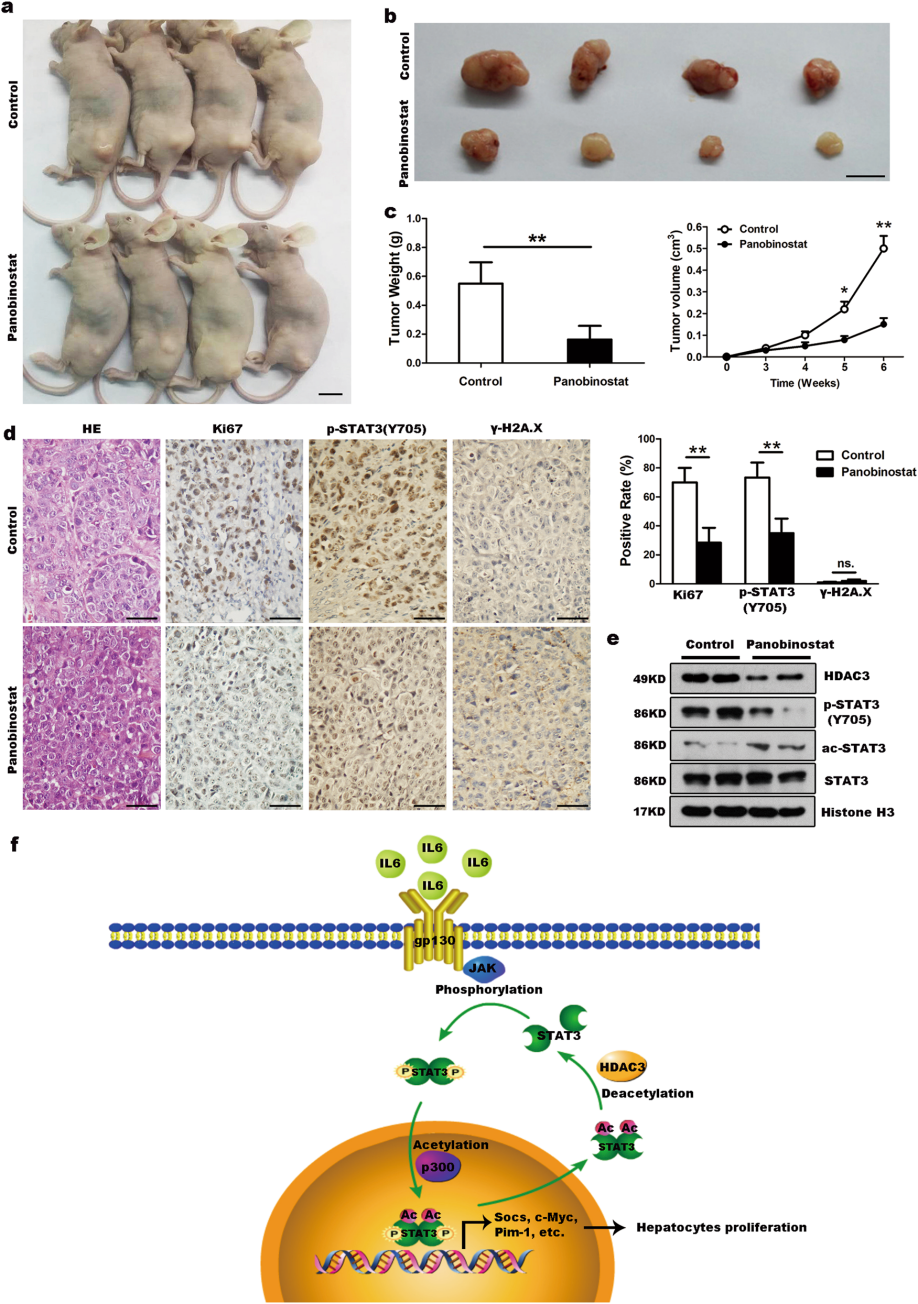
Inhibition of HDAC3 expression decreases HCC xenografts growth. **a–c** HCC growth in mouse xenograft model. SMMC-7721 cells (2 × 10^6^) were injected subcutaneously into nude mice for xenograft assay. Tumor volume and average weight of HCC xenografts in each group were shown (*n* = 4) (scale bar: 1 cm). **d** Immunohistochemical analysis of control and panobinostat-treated tumors with p-STAT3(Y705), Ki-67, and γ-H2A.X antibodies (scale bar: 50 μm). Quantitative analysis of p-STAT3(Y705), Ki-67, and γ-H2A.X-positive rates in each group of tumors were shown. **e** Western blot analysis demonstrates that p-STAT3(Y705) is inhibited in panobinostat-treated tumors. Histone H3 were used as the loading controls. **f** Diagram of the HDAC3–STAT3 axis in the IL6/STAT3 signaling pathway and the signaling cascades that regulate hepatocyte proliferation and tumorigenesis. All data represent the mean ± SD; *p < 0.05; **p < 0.01; n.s. not significant

To elucidate the effect of elevated HDAC3 on HCC growth, SMMC-7721 cells were injected subcutaneously into nude mice for xenograft assay. We treated xenograft with panobinostat, a pan-HDAC-inhibitor for clinical treatment of lymphoma and multiple myeloma^12^, to reduce HDAC3 expression in SMMC-7721 cells. HDAC3 inhibition significantly reduced tumor growth and decreased the expression of Ki67 in HCC xenograft tumors (Fig. 7a–d). Consistently, the expression of p-STAT3(Y705) in panobinostat-treated xenograft tumors was significantly reduced (Fig. 7d, e). In addition, HDAC3 knockdown remarkably suppressed cell cycle progression and inhibited the phosphorylation of STAT3(Y705) (Supplementary Fig. 8). Therefore, increased HDAC3 enhanced tumor growth that may occur through STAT3 signaling in liver cancer cells.

## Discussion

HDAC3 is a crucial deacetylase component of the NCOR1/SMRT complexes, which is involved in histone modifications, chromatin remodeling, and transcriptional regulation^5,37^. In addition to the important role of HDAC3 in DNA repair and in the maintenance of the circadian rhythm of metabolism homeostasis, the essential non-transcriptional function of HDAC3 during mitosis is still largely unknown. Here, we have established for the first time the importance of HDAC3 for liver regeneration together with liver cancer cells proliferation.

Two types of transmembrane enzyme-linked receptor-mediated signaling cascades, namely, the cytokine-dependent pathway and the growth factor-dependent pathway, which transduce extracellular growth stimuli into the nucleus, play crucial roles in priming liver regeneration^1,23^. Our previous study showed that loss of the stimulatory G protein α subunit (G_s_α), which activates the cAMP-dependent pathway, leads to a breakdown in growth factor signaling and dramatically arrests the regenerating hepatocytes in the G1 phase extension^38^. Lack of HDAC3 results in a similar proliferative defect at the cell cycle entrance, which indicates a breakdown in the cytokine-dependent or growth factor-dependent signaling cascade. Using a microarray assay analysis and LPS/PH model, we showed that HDAC3 deficiency dramatically impairs the STAT3 signaling pathway, which is a critical signaling cascade that promotes the transcription of numerous immediate-early genes in response to acute liver injury.

As a nucleocytoplasmic shuttling transcription factor, STAT3 is acetylated by p300 in the nucleus^39^; however, the details regarding deacetylation of STAT3 remain unclear. Our data show that HDAC3 deficiency remarkably increases the p300-dependent STAT3 acetylation in the cytoplasm of hepatocytes. It has been suggested that ac-STAT3 is likely to be deacetylated by class I HDAC members in the nucleus^28^. Unlike HDAC1 or HDAC2, which predominantly function in the nucleus, HDAC3 is a unique Class I HDAC subfamily member that regulates acetylation status of non-histone proteins in the cytoplasm^33,34^. Our results revealed that HDAC1 and/or HDAC2 deficiency does not change the acetylation status of STAT3 in the liver, while loss of HDAC3 results in a substantial cytoplasmic accumulation of ac-STAT3, indicating that the deacetylation of ac-STAT3 might be selectively dependent on HDAC3 in the cytoplasm rather than in the nucleus.

After IL6 stimulation, STAT3 that resides in the cytoplasm rapidly becomes phosphorylated and translocates into the nucleus to induce target genes expression^24^. Our data show that HDAC3 deficiency significantly increases ac-STAT3 level and decreases p-STAT3(Y705) level together with a decrease in STAT3 nucleus entry, suggesting that HDAC3-mediated deacetylation of ac-STAT3 is essential for STAT3(Y705) phosphorylation and STAT3 reactivation cycle. It has been shown that the conserved lysine residues for acetylation in STAT3 are all near the Y705 residual of STAT3^29^, supporting the possibility that STAT3 acetylation induced by HDAC3 deficiency could prevent STAT3(Y705) phosphorylation and block STAT3 signaling activation. However, the detail mechanisms of HDAC3-mediated modifications of STAT3 deacetylation and phosphorylation in hepatocytes remain further studies. Regardless of other mechanisms that might be regulated by HDAC3 during the whole process of hepatocyte proliferation, HDAC3 is undoubtedly critical to the initiation of the cell cycle progression through the proper transition of ac-STAT3 to p-STAT3. Therefore, HDAC3 may act as a molecular switch in the STAT3 signaling cascade that transmits growth stimuli from the cytoplasm to the nucleus.

Multiple studies have shown that HDAC3 is aberrantly expressed in various human cancers and that the aberrant expression is often associated with a poor prognosis^37^, making HDAC3 inhibition a potential strategy for cancer therapy. The HDAC3 mRNA level is lacked in one-sixth of human HCC tissues^11^. The inactivation of HDAC3 may contribute to HCC development by increasing global histone acetylation, defective DNA damage repair, and mutation accumulation^11^. HDAC3 is also upregulated in a specific subset of HCCs^12,13^; however, the role of HDAC3 in the pathogenesis of HCC remains unknown. The upregulated expression of HDAC3 in human HCC prompts us to determine whether the HDAC3–STAT3 pathway enhances HCC tumor growth. Notably, IL6, a cytokine that is essential for activating STAT3 signaling in liver inflammation, enhances HCC development^36^. We found that overexpression of HDAC3 is significantly associated with an increased p-STAT3(Y705) level and Ki67 index in HCC. Moreover, our xenograft assay shows that a low dose of panobinostat could significantly decrease HCC xenografts growth along with a significant reduction in p-STAT3(Y705) level. Our data suggest that the drug panobinostat approved by FDA might also prevent liver cancer cells proliferation through HDAC3–STAT3 signaling pathway in HDAC3-overexpressed HCC, while the detail effects and mechanisms of panobinostat for the clinical treatment of HCC remain further studies.

In summary, we have demonstrated that HDAC3 acts an essential role in the enhancement of the STAT3 signaling pathway by catalyzing the deacetylation of ac-STAT3 in the cytoplasm, thereby, promoting liver regeneration and tumor growth, as illustrated in Fig. 7f. Our findings highlight the importance of the HDAC3–STAT3 signaling cascade in promoting cell cycle progression and support HDAC3 as a potential therapeutic target for the treatment of HCC as well as other solid cancers whose HDAC3 is overexpressed.

## Materials and methods

### Mice

All mouse experimental procedures were approved by the Animal Care and Use Committee of Sichuan University. HDAC3^loxP/loxP^ frozen embryos were purchased from the European Mouse Mutant Archive (EMMA), University of Veterinary Medicine, Vienna. Alb-Cre and Alb-Cre^ERT2^ transgenic mice were purchased from Shanghai Biomodel Organism Science & Technology Development Co., Ltd, China. The genotypes of Alb-Cre;HDAC3^loxP/loxP^ and Alb-Cre^ERT2^;HDAC3^loxP/loxP^ mice were determined using PCR amplification of tail DNA. The sequences of genotyping primers are presented in Supplementary Table 1.

For inducible deletion of HDAC3, tamoxifen was dissolved in corn oil containing 10% (vol/vol) ethanol to 20 mg/ml and shaken overnight at 37 °C^20^. Six-week-old male Alb-Cre^ERT2^;HDAC3^loxP/loxP^ mice were injected intraperitoneally with tamoxifen (2 mg per mouse) for five consecutive days. Experiments on mice were performed 5 days after the last injection. Then, these mice were treated with either classical 2/3 PH surgery or intraperitoneal injection of CCl_4_ (10 ml/kg body weight)^31^. BrdU (1 mg/kg body weight) was intraperitoneally injected 60 min before sacrifice.

### Statistical analysis

Data were expressed as the mean ± standard deviation (SD). Statistical comparisons were assessed with an unpaired, one-tailed Student’s *t-*test with Welch correction. A *p-*value < 0.05 was considered significant.

## Electronic supplementary material

Supplemental Information(DOCX 39 kb)

Supplementary Figure1(JPG 2635 kb)

Supplementary Figure2(JPG 2619 kb)

Supplementary Figure3(JPG 2374 kb)

Supplementary Figure4(JPG 5649 kb)

Supplementary Figure5(JPG 2727 kb)

Supplementary Figure6(JPG 1569 kb)

Supplementary Figure7(JPG 3396 kb)

Supplementary Figure8(JPG 4006 kb)

Supplementary Figure9(JPG 2420 kb)

Supplementary Figure10(JPG 2481 kb)
