# Supplementary material for: Histone deacetylase 3 promotes liver regeneration and liver cancer cells proliferation through signal transducer and activator of transcription 3 signaling pathway
Source: Cell Death Dis. 2018 Mar 14;9(3):398. doi: 10.1038/s41419-018-0428-x (PMC5852132; doi:10.1038/s41419-018-0428-x)
Supplement: Supplementary file 1 — Supplemental Information(DOCX 39 kb) [file 41419_2018_428_MOESM1_ESM.docx]

**­Histone Deacetylase 3 Promotes Liver Regeneration and Liver Cancer Cells Proliferation through Signal Transducer and Activator of Transcription 3** **Signaling Pathway**

Xu-Feng Lu^1,†^, Xiao-Yue Cao^1,†^, Yong-Jie Zhu^1^, Zhen-Ru Wu^1^, Xiang Zhuang^1,2^, Ming-Yang Shao^1^, Qing Xu^1^, Yong-Jie Zhou^1^, Hong-Jie Ji^1^, Qing-Richard Lu^3^, Yu-Jun Shi^1,*^, Yong Zeng^4^, and Hong Bu^1, 2^

1 Laboratory of Pathology, Key Laboratory of Transplant Engineering and Immunology, NHFPC; West China Hospital, Sichuan University, Chengdu 610041, China.

2 Department of Pathology, West China Hospital, Sichuan University, Chengdu 610041, China.

3 Department of Pediatrics, Division of Experimental Hematology and Cancer Biology, Brain Tumor Center, Cincinnati Children's Hospital Medical Center, Cincinnati, Ohio 25229, USA.

4 Department of Liver and Vascular Surgery, West China Hospital, Sichuan University, Chengdu 610041, China.

† The authors contributed equally to this work.

* **Corresponding Author**: Yujun Shi, PhD, MD, Laboratory of Pathology, West China Hospital, Sichuan University, 37 Guoxue Road, Chengdu 610041, China, E-mail: **shiyujun@scu.edu.cn**. **Telephone/Fax:** +86-028-85164050.

**Supplementary Fig. 1. Impaired liver metabolism and severe DNA damage in the Alb-Cre; HDAC3^loxP/loxP^ mouse liver.**

(a) The electropherogram of the tail DNA amplified by PCR is shown. The β-actin gene was used as a positive control for the Cre gene.

(b) Western blot analysis of the HDAC3, H3K9ac and H4K16ac expression levels. Histone H3 was used as the loading controls.

(c) The liver morphology, HE staining, glutamine synthetase (GS) immunohistochemistry, periodic acid–schiff (PAS) staining and Oil Red O staining show a disrupted liver architecture and dysregulated hepatic metabolism in the Alb-Cre;HDAC3^loxP/loxP^ mouse liver.

(d) Immunohistochemistry for γ-H2A.X (a marker of DNA double-stand breaks) in the Alb-Cre;HDAC3^loxP/loxP^ mouse liver.

(e) The γ-H2A.X-positive hepatocyte index and the upregulated serum AST and ALT levels in Alb-Cre; HDAC3^loxP/loxP^ mice. All data represent the mean ± SD; n = 3-6; **p < 0.01; ***p < 0.001. Scale bar: 50 μm.

**Supplementary Fig. 2. The inducible short-term absence of hepatic HDAC3 did not obviously alter hepatic metabolism and DNA stability in the HDAC3^△^ mouse liver.**

(a) Schematic of the experimental protocol for establishing the tamoxifen-dependent hepatocyte HDAC3 deletion and the subsequent experiment analyses.

(b) Western blot analysis of the HDAC3 knockout efficiency and the H3K9ac and H4K16ac expression levels in the HDAC3^△^ liver. Histone H3 was used as the loading controls.

(c) The liver morphology, HE staining, GS immunohistochemistry, PAS staining and Oil Red O staining show a normal liver architecture and hepatic metabolism in the HDAC3^△^ mouse liver.

(d) Immunohistochemistry for γ-H2A.X in the HDAC3^△^ mouse liver.

(e) The γ-H2A.X-positive hepatocyte index and the normal serum AST and ALT levels in the HDAC3^△^ mice. All data represent the mean ± SD; n = 3-8; ***p < 0.001; ns. not significant. Scale bar: 50 μm.

**Supplementary Fig. 3. HDAC3 deficiency delays liver regeneration.**

(a) Immunohistochemistry of Ki67 confirms continuous liver regeneration in the HDAC3^△^ mice at 14 d after PH.

(b) The relative protein levels of CDK1, CDK2, CDK4, cyclinA2, cyclinB1, cyclin D1, cyclin E1 and p-H3S10 at the indicated time points after 70% PH are shown. All data represent the mean ± SD; n = 3-8; **p < 0.01; ***p < 0.001. Scale bar: 50 μm.

**Supplementary Fig. 4. Delayed liver repair in HDAC3^△^ mice following CCl4 challenge.**

(a) HE staining shows that liver regeneration is dramatically impaired in the HDAC3^△^ mice after the CCl4 treatment. Necrotic zones are circled with black-dotted lines (scale bar: 100 μm).

(b, c) Immunohistochemistry to assess BrdU and Ki67 at the indicated time points following the CCl4 challenge (scale bar: 50 μm).

(d) Serum AST and ALT levels at the indicated time points after the CCl4 treatment.

(e) Serum IL6 levels at the indicated time points after the CCl4 treatment.

(f, g) Western blot analysis of p-STAT3(Y705), c-myc and cyclin D1 following the CCl4 challenge. GAPDH was used as the loading control. All data represent the mean ± SD; n = 3-5; *p < 0.05; **p < 0.01; ***p < 0.001.

**Supplementary Fig. 5. Microarray analysis demonstrates the modest effects of HDAC3 on gene expression after the tamoxifen** **treatment.**

(a) A heat map of the 172 differentially expressed genes (DEGs; fold changes ≥ 1.5 and p < 0.05) in the control and mutant livers after the tamoxifen injections.

(b) The number of changed genes in the HDAC3^△^ livers after the tamoxifen treatment.

(c) GO term analysis of the list of DEGs (fold changes ≥ 1.5 and p < 0.05) indicated that metabolic pathways are slightly changed in the HDAC3^△^ mice before PH.

(d) qRT-PCR analysis of the cell cycle genes in the control and HDAC3^△^ livers. All data represent the mean ± SD; n = 3; **p < 0.01; ***p < 0.001; ns. not significant.

**Supplementary Fig. 6. C-myc remains inhibited in mutant hepatocytes after LPS/PH treatment.**

(a) Serum IL6 level and the relative protein level of p-STAT3(Y705) and c-myc at the indicated time points after PH.

(b) Schematic of the experimental protocol for LPS/PH model.

(c) Serum IL6 level and the relative protein level of p-STAT3(Y705) and c-myc at the indicated time points after LPS/PH. All data represent the mean ± SD; n = 3-5; *p < 0.05; **p < 0.01; ***p < 0.001.

**Supplementary Fig. 7. Primary diploid hepatocyte isolation and sorting.**

(a) Schematic diagram of the construction of the dual reporter system of the Alb-Cre^ERT2^;HDAC3^loxP/loxP^;mT/mG mice.

(b) Schematic of the lineage tracing experiments of the primary diploid hepatocyte isolation and sorting (scale bar: 50 μm).

**Supplementary Fig. 8. HDAC3 knockdown inhibits STAT3(Y705) phosphorylation in HepG2 cells.**

(a) HDAC3 knockdown increased ac-STAT3 level in HepG2 cells.

(b) HDAC3 knockdown inhibits STAT3(Y705) phosphorylation due to the high level of ac-STAT3 in HepG2 cells after IL6 treatment. β-actin was used as the loading controls.

(c) Flow cytometric analysis of cell cycle distributions of HepG2 cells after siRNA transfection.

(d) Immunofluorescence demonstrates that STAT3(Y705) fails to be phosphorylated in HDAC3 knockdown cells after IL6 treatment (scale bar: 10 μm).

(e) Co-immunoprecipitation assays demonstrate that HDAC3 combines with ac-STAT3 in the cytoplasm. All data represent the mean ± SD; n = 3.

**Supplementary Fig. 9. HDAC8 deficiency does not affect liver regeneration.**

(a-c) Immunohistochemistry of BrdU and Ki67 confirms that loss of HDAC8 has a negligible effect on liver regeneration after PH. All data represent the mean ± SD; n = 3-6; ns. not significant. Scale bar: 50 μm.

**Supplementary Fig. 10. HDAC3 with combined Ki67 expression reduces the overall survival rate of HCC patients.**

(a) Representative microphotograph of Ki67 expression in HCC tissues (n = 90) by immunohistochemistry (scale bar: 50 μm).

(b) Statistical analysis of immunohistochemistry-based Ki67 expression in the normal liver (n = 7), tumor and corresponding nontumor tissues (n = 90).

(c) Kaplan-Meier analysis shows that single upregulation of Ki67 (n = 90) or combined upregulation of HDAC3 and Ki67 (n = 84) significantly reduces the overall survival rate of HCC patients. All data represent the mean ± SD; ***p < 0.001.

**Supplementary Table 1. Genotyping primers for conditional knockout mice**.

| Gene | Gene ID | Primer Sequence |
| --- | --- | --- |
| HDAC3 | 15183 | GCTTGGTAGCCAGCCAGCTTAG |
|  |  | CATGTGACCCCAGACATGACTGG |
|  |  | CAGTCCATGCCTATAATCCCAGC |
| Cre | 2777477 | CACCCTGTTACGTATAGCCG |
|  |  | GAGTCATCCTTAGCGCCGTA |
| β-actin | 11461 | CCTAGGCACCAGGGTGTGAT |
|  |  | TCACGGTTGGCCTTAGGGTT |

**Supplementary Table 2. Antibodies and reagents used in the study.**

| **Antibody/ Reagent** | **Company** | **Catalog#:** |
| --- | --- | --- |
| BrdU | Thermo Fisher Scientific | MS-1058-P0 |
| Ki67 | Thermo Fisher Scientific | RM-9106-S1 |
| pH3S10 | Merck Millipore | 06-570 |
| Glutamine Synthetase | Abcam | ab49873 |
| Cyclin A2 | Santa Cruz Biotechnology | sc-751 |
| Cyclin B1 | Cell Signaling Technology | 4138 |
| Cyclin D1 | Abcam | ab134175 |
| Cyclin E1 | Abcam | ab88259 |
| CDK1 | Abcam | ab32384 |
| CDK2 | Cell Signaling Technology | 2546 |
| CDK4 | Abcam | ab199728 |
| p-STAT3(Y705) | Cell Signaling Technology | 9145 |
| p-STAT3(S727) | Cell Signaling Technology | 94994 |
| ac-STAT3 | Cell Signaling Technology | 2523 |
| STAT3 | Cell Signaling Technology | 9139 |
| p300 | Cell Signaling Technology | 7389 |
| JAK2 | Cell Signaling Technology | 3230 |
| p-JAK2 | Cell Signaling Technology | 3776 |
| c-myc | Abcam | ab32072 |
| HDAC1 | Cell Signaling Technology | 34589 |
| HDAC2 | Abcam | ab32117 |
| HDAC3 | Cell Signaling Technology | 3949 |
| HDAC3 | Novus Biologicals | NBP1-19396 |
| Histone H3 (acetyl K9) | Abcam | ab32129 |
| Histone H4 (acetyl K16) | Abcam | ab109463 |
| gamma H2A.X (phospho S139) | Abcam | ab2893 |
| β-Actin | Cell Signaling Technology | 8457 |
| Histone H3 | Cell Signaling Technology | 9715 |
| GAPDH | KangChen Bio-tech | KC-5G4 |
| Donkey anti-Mouse IgG (H+L), Alexa Fluor 488 | Thermo Fisher Scientific | A-21202 |
| Goat anti-Rabbit IgG (H+L), Alexa Fluor 488 | Thermo Fisher Scientific | A-11070 |
| Dako REAL™ EnVision™ Detection System | Dako | k5007 |
| iScript cDNA synthesis kit | Bio-Rad | 179-8890 |
| SsoFast^TM^ EvaGreen Supermix | Bio-Rad | 172-5201AP |
| chemiluminescence reagent | PerkinElmer | NEL104001EA |
| NE-PER™ Nuclear and Cytoplasmic Extraction Reagents | Thermo Fisher Scientific | 78833 |
| Reversible Immunoprecipitation System kit | Merck Millipore | 17-500 |
| Tamoxifen | Sigma-Aldrich | T5648 |
| 5-bromo-2-deoxyuridine | Sigma-Aldrich | B5002 |
| DAPI | Sigma-Aldrich | D9542 |
| TRIzol reagent | Thermo Fisher Scientific | 15596018 |
| phosphatase inhibitor cocktail | Sigma-Aldrich | P5726 |
| protease inhibitor cocktail | Sigma-Aldrich | P8340 |
| DMEM, high glucose | Gibco | 11995040 |
| fetal bovine serum | Gibco | 10099141 |
| penicillin/streptomycin | Gibco | 15070063 |
| EGF | Sigma-Aldrich | E4127 |
| Insulin | Thermo Fisher Scientific | 51500056 |
| IL6 | Sigma-Aldrich | SRP3330 |
| Lipofectamine® 2000 | Thermo Fisher Scientific | 11668027 |
| Cell Counting Kit-8 | Dojindo | CK04 |
| Panobinostat(LBH589) | MedChemExpress | HY-10224 |

**Supplementary Table 3. Primers for qRT-PCR.**

| Gene | Gene ID | Primer Sequence |
| --- | --- | --- |
| c-fos | 14281 | CGGGTTTCAACGCCGACTA |
|  |  | TGGCACTAGAGACGGACAGAT |
| c-jun | 16476 | TTCCTCCAGTCCGAGAGCG |
|  |  | TGAGAAGGTCCGAGTTCTTGG |
| Egr2 | 13654 | CCGTATCCGAGTAGCTTCGC |
|  |  | TCAATGGAGAATTTGCCCATGT |
| Egr3 | 13655 | TTGCCTGACAATCTGTACCCC |
|  |  | TAATGGGCTACCGAGTCGCT |
| Gadd45a | 13197 | AGACCGAAAGGATGGACACG |
|  |  | GTACACGCCGACCGTAATG |
| Junb | 16477 | TCACGACGACTCTTACGCAG |
|  |  | CCTTGAGACCCCGATAGGGA |
| Mdm2 | 17246 | TAAAGTCCGTTGGAGCGCAAA |
|  |  | CTGCTGCTTCTCGTCATATAACC |
| SOCS3 | 12702 | TGCGCCTCAAGACCTTCAG |
|  |  | GCTCCAGTAGAATCCGCTCTC |
| STAT3 | 20848 | CACCTTGGATTGAGAGTCAAGAC |
|  |  | AGGAATCGGCTATATTGCTGGT |
| c-myc | 17869 | ATGCCCCTCAACGTGAACTTC |
|  |  | GTCGCAGATGAAATAGGGCTG |
| Cyclin D1 | 12443 | GCGTACCCTGACACCAATCTC |
|  |  | ACTTGAAGTAAGATACGGAGGGC |
| Cyclin E1 | 12447 | CTCCGACCTTTCAGTCCGC |
|  |  | CACAGTCTTGTCAATCTTGGCA |
| CDK1 | 12534 | AGGTACTTACGGTGTGGTGTAT |
|  |  | CTCGCTTTCAAGTCTGATCTTCT |
| CDK2 | 12566 | ATGGAGAACTTCCAAAAGGTGG |
|  |  | CAGTCTCAGTGTCGAGCCG |
| CDK4 | 12567 | TCAGCACAGTTCGTGAGGTG |
|  |  | TCCATCAGCCGTACAACATTG |
| Cyclin A2 | 12428 | AAGAGAATGTCAACCCCGAAAAA |
|  |  | ACCCGTCGAGTCTTGAGCTT |
| Cyclin B1 | 268697 | GCGTGTGCCTGTGACAGTTA |
|  |  | CCTAGCGTTTTTGCTTCCCTT |
| Cdkn1a(p21) | 12575 | CCTGGTGATGTCCGACCTG |
|  |  | CCATGAGCGCATCGCAATC |
| Cdkn1b(p27) | 12576 | TCAAACGTGAGAGTGTCTAACG |
|  |  | CCGGGCCGAAGAGATTTCTG |
| Cdkn2a(p16) | 12578 | ACATCAAGACATCGTGCGATATT |
|  |  | CCAGCGGTACACAAAGACCA |
| Cdkn2b(p15) | 12579 | CCCTGCCACCCTTACCAGA |
|  |  | GCAGATACCTCGCAATGTCAC |
| β-actin | 11461 | GTGACGTTGACATCCGTAAAGA |
|  |  | GCCGGACTCATCGTACTCC |

**Supplementary Table 4. Relationship of HDAC3, p-STAT3(Y705) and Ki67 expression level with the clinicopathological features in HCC.**

| Variables | N | HDAC3 expression | |  | p-STAT3(Y705) expression | |  | Ki67 expression | |  |
| --- | --- | --- | --- | --- | --- | --- | --- | --- | --- | --- |
|  | 90 | Negative  N=63 | Positive  N=27 | p | Low  (≤50%)  N=37 | High  (>50%)  N=53 | p | Low  (≤50%)  N=69 | High  (>50%)  N=21 | p |
| Age |  |  |  | 0.268 |  |  | 0.457 |  |  | 0.272 |
| ≤50 | 42 | 27 | 15 |  | 19 | 23 |  | 30 | 12 |  |
| >50 | 48 | 36 | 12 |  | 18 | 30 |  | 39 | 9 |  |
| Gender |  |  |  | 0.470 |  |  | 0.175 |  |  | 0.409 |
| Male | 74 | 53 | 21 |  | 28 | 46 |  | 58 | 16 |  |
| Female | 16 | 10 | 6 |  | 9 | 7 |  | 11 | 5 |  |
| AFP |  |  |  | 0.340 |  |  | 0.674 |  |  | 0.870 |
| ≤20 | 27 | 17 | 10 |  | 12 | 15 |  | 21 | 6 |  |
| >20 | 63 | 46 | 17 |  | 25 | 38 |  | 48 | 15 |  |
| Cirrhosis |  |  |  | 0.343 |  |  | 0.966 |  |  | 0.379 |
| Yes | 78 | 56 | 22 |  | 32 | 46 |  | 61 | 17 |  |
| No | 12 | 7 | 5 |  | 5 | 7 |  | 8 | 4 |  |
| HBsAg |  |  |  | 0.472 |  |  | 0.413 |  |  | 0.981 |
| Yes | 77 | 55 | 22 |  | 33 | 44 |  | 59 | 18 |  |
| No | 13 | 8 | 5 |  | 4 | 9 |  | 10 | 3 |  |
| Tumor size |  |  |  | 0.117 |  |  | 0.067 |  |  | 0.110 |
| ≤5 cm | 48 | 37 | 11 |  | 24 | 24 |  | 40 | 8 |  |
| >5 cm | 42 | 26 | 16 |  | 13 | 29 |  | 29 | 13 |  |
| Tumor multiplicity |  |  |  | 0.904 |  |  | 0.377 |  |  | 0.409 |
| Single | 74 | 52 | 22 |  | 32 | 42 |  | 58 | 16 |  |
| Multiple | 16 | 11 | 5 |  | 5 | 11 |  | 11 | 5 |  |
| TNM Stage |  |  |  | 0.258 |  |  | 0.180 |  |  | 0.234 |
| Ⅰ-Ⅱ | 61 | 45 | 16 |  | 28 | 33 |  | 49 | 12 |  |
| Ⅲ-Ⅳ | 29 | 18 | 11 |  | 9 | 20 |  | 20 | 9 |  |
| Histopathologic  Grade |  |  |  | 1.000 |  |  | 0.545 |  |  | 1.000 |
| Ⅰ-Ⅱ | 60 | 42 | 18 |  | 26 | 34 |  | 46 | 14 |  |
| Ⅲ | 30 | 21 | 9 |  | 11 | 19 |  | 23 | 7 |  |
| Recurrence |  |  |  | 0.006 |  |  | 0.099 |  |  | 0.231 |
| Present | 53 | 43 | 10 |  | 18 | 35 |  | 43 | 10 |  |
| Absent | 37 | 20 | 17 |  | 19 | 18 |  | 26 | 11 |  |

**Supplementary Table 5. Univariate analyses of factors associated with overall survival (OS) and time to recurrence (TTR) in primary HCC cohort.**

| Variables | OS Relative risk | 95% CI | p | TTR Relative risk | 95% CI | p |
| --- | --- | --- | --- | --- | --- | --- |
| Age (year) (>50 vs. ≤50) | 0.831 | 0.446-1.549 | 0.560 | 0.797 | 0.428-1.485 | 0.475 |
| Gender (male vs. female) | 1.764 | 0.748-4.158 | 0.195 | 1.808 | 0.764-4.276 | 0.178 |
| AFP (ng/ml) (>20 vs. ≤20) | 1.108 | 0.535-2.294 | 0.783 | 1.145 | 0.552-2.373 | 0.717 |
| Cirrhosis (yes vs. no) | 1.063 | 0.394-2.873 | 0.904 | 1.074 | 0.397-2.909 | 0.888 |
| HBsAg (yes vs. no) | 1.184 | 0.431-3.255 | 0.743 | 1.224 | 0.444-3.376 | 0.696 |
| Tumor size (cm) (>5 vs. ≤5) | 1.616 | 0.825-3.162 | 0.161 | 0.611 | 0.313-1.192 | 0.149 |
| Tumor multiplicity (multiple vs. single) | 1.196 | 0.558-2.567 | 0.645 | 0.791 | 0.369-1.698 | 0.548 |
| TNM Stage (Ⅰ-Ⅱ vs. Ⅲ-Ⅳ) | 0.837 | 0.444-1.579 | 0.583 | 0.873 | 0.464-1.643 | 0.673 |
| Histopathologic  Grade (Ⅰ-Ⅱ vs. Ⅲ) | 0.787 | 0.391-1.586 | 0.503 | 1.217 | 0.607-2.440 | 0.580 |
| Recurrence (present vs. absent) | 8.786 | 3.768-20.486 | 0.000 | 8.738 | 3.738-20.428 | 0.000 |
| HDAC3 expression (positive vs. negative) | 3.893 | 1.766-8.581 | 0.000 | 3.806 | 1.719-8.426 | 0.000 |
| p-STAT3 expression (low vs. high) | 0.463 | 0.238-0.899 | 0.023 | 0.451 | 0.232-0.876 | 0.019 |
| Ki67 expression (high vs. low) | 4.582 | 0.841-24.975 | 0.078 | 4.415 | 0.812-24.003 | 0.086 |

**Supplementary Table 6. Independent prognostic factors for OS and TTR by multivariate analyses in primary and validation cohort.**

| Variables | OS Relative risk | 95% CI | p | TTR Relative risk | 95% CI | p |
| --- | --- | --- | --- | --- | --- | --- |
| Recurrence (present vs. absent) | 10.037 | 4.410-22.844 | 0.000 | 9.698 | 4.281-21.969 | 0.000 |
| HDAC3 expression (positive vs. negative) | 4.895 | 2.446-9.794 | 0.000 | 4.646 | 2.334-9.247 | 0.000 |
| p-STAT3 expression (low vs. high) | 0.530 | 0.302-0.931 | 0.027 | 0.518 | 0.295-0.912 | 0.023 |

**Supplemental Material**

**Methods**

**Immunohistochemistry and immunofluorescence**

Liver samples were fixed in 10% neutral buffered formalin for 24-36 h. Sections were performed with a series of dewaxing, rehydration, antigen retrieval, and quenching of endogenous peroxidase activity. After blocking with 5% goat serum, the sections were labeled with corresponding primary antibody at 4°C overnight and anti-mouse/rabbit secondary antibody (Dako REAL™ EnVision™ Detection System) for 1 h at room temperature. For immunohistochemistry, detection was developed by 3,3’-diaminobenzidine (DAB) substrate. For immunofluorescence, Alexa fluorescently labeled secondary antibodies were used at a 1:300 dilution. Nuclear DNA was counterstained with 100 nM DAPI. The antibodies and reagents used are listed in Supplementary Table 2.

**Western blotting**

Cells and liver tissues were lysed in RIPA buffer and homogenized with FastPrep®-24 (MP Biomedicals, Illkirch, France). Proteins were separated on a polyvinylidene membrane by sodium dodecyl sulfate polyacrylamide gel electrophoresis (SDS-PAGE). The membrane was incubated with primary antibody in TBST containing 5% nonfat milk overnight at 4°C, and subsequently incubated with the corresponding horseradish peroxidase-conjugated secondary antibody. A chemiluminescence reagent was used for chemiluminescence detection of protein levels. Western blots were quantified with Image J software (National Institutes of Health, Bethesda, MD). The antibodies and reagents used are listed in Supplementary Table 2.

**Immunoprecipitation**

Liver tissues were lysed in RIPA buffer supplemented with a mixture of a phosphatase inhibitor and protease inhibitor cocktail. The protein concentration was quantified by the NanoDrop Microvolume Spectrophotometer (Thermo Scientific, Wilmington, DE). The indicated antibodies were used to incubate with protein samples at 4 °C overnight according to manufacturer’s instructions of Reversible Immunoprecipitation System kit. After rinsing the protein G or A Sepharose beads with lysis buffer, supernatants were subsequently used for Western blotting. The antibodies and reagents used in this study are listed in Supplementary Table 2.

**Primary hepatocyte isolation and cell culture**

Primary hepatocytes were isolated from 7-week-old mice using collagenase perfusion method as described.^1^ Diploid hepatocytes were separated by fluorescence activated cell sorting (FACS) as described.^2^ Primary hepatocytes were seeded on type I collagen-coated dish (Biocoat, BD Biosciences) at a density of 3.0×10^4^/cm^2^ in high glucose Dulbecco’s modified Eagle’s medium (DMEM) containing 10% fetal bovine serum (FBS), 2 mM L-glutamine and 100 U/mL of penicillin/streptomycin. For the experiments, primary hepatocytes at 60% confluence were serum-starved overnight and were treated with 20 ng/mL IL6 or an equivalent volume of PBS. The reagents used are listed in Supplementary Table 2.

**Analysis of cell viability of primary hepatocytes.**

Primary hepatocytes seeded on 6-well collagen-coated plates at 60% confluence were serum-starved overnight and were stimulated with 10 ng/mL of epidermal growth factor (EGF) and 20 mIU/mL of insulin, and cell viability was performed by trypan blue staining.^1^ In each experiment, cells were counted in a Neubauer chamber, cell number was determined based on three technical replicates.

**qRT-PCR**

Total RNA was isolated from liver samples using TRIzol reagent. cDNA synthesis was performed using the iScript cDNA synthesis kit. CFX96^TM^ Real-Time PCR system (Bio-Rad, Hercules, CA) and SsoFast^TM^ EvaGreen Supermix was used for q-PCR reactions. mRNA levels of target genes were normalized to β-actin gene expression, relative gene expression levels were analyzed using the △△t method. Relative genes expression with a greater than 2-fold change was considered statistically significant. The sequences of the indicated primers are presented in Supplementary Table 3.

**Human clinical samples**

Fresh and paraffin-fixed tumor specimens were collected from HCC patients who underwent curative resection in the West China Hospital, Sichuan University. Human samples were obtained from all patients with written informed consent. The procedures for human sample collection and the use of human samples were approved by the ethics committee of the West China Hospital, Sichuan University (Chengdu, China). Tissue array blocks containing HCC tissues and their corresponding non-HCC tissues (n=90) were generated with a tissue microarrayer (Beecher Instruments, Silver Spring, MD). Survival analysis was determined by Kaplan-Meier followed by log-rank test. The distribution of the clinicopathologic data in this study cohort is given in Supplementary Table 4.

**The Cancer Genome Atlas**

Transcriptome sequencing data and patient survival data of 373 HCC patients were obtained from The Cancer Genome Atlas (TCGA) via the Broad GDAC Firehose (Broad Institute). HDAC3 expression in HCC patients was defined as high if the Z score > 1, or otherwise low.^3^

**Nuclear and cytoplasmic extraction**

Nuclear and cytoplasmic extracts were separated according to manufacturer’s instructions of NE-PER™ Nuclear and Cytoplasmic Extraction Reagents.

**LPS/PH experiment**

Liver removal was used as time 0 during subsequent experiments. LPS (4 mg/kg) (Escherichia coli O55:B5; Sigma) was injected intraperitoneally soon after PH.^4^

**Serum biochemistry and cytokine assay**

Blood samples were obtained from the orbital vascular plexus of the mice. Serum levels of AST and ALT were determined. Serum levels of cytokines were analyzed on a Luminex 100 system using a Millipore Map Mouse Cytokine/Chemokine Magnetic Bead Panel kit (Millipore Corporation, Billerica, MA) according to the manufacturer’s instructions.

**Microarray analysis**

RNA was extracted from mouse livers using an RNeasy Microarray Tissue Mini Kit (QIAGEN). Total RNA was qualified using an Agilent 2100 Bioanalyzer and prepared for sequencing using the Illumina RNA-sequencing kit according to the manufacturer's instructions. RNA-sequencing were performed by the Beijing Genomics Institute (BGI, China). The Database for Annotation, Visualization and Integrated Discovery (DAVID) was used to determine overrepresented gene ontology (GO) categories, using the entire mouse transcriptome as the background gene set.

**Cell culture and siRNA transfection**

The human liver cancer cell HepG2 was maintained in Dulbecco’s modified Eagle’s medium (DMEM) supplemented with 10% fetal bovine serum, 2 mM/L glutamine, 100 U/ml penicillin/streptomycin. The cells were maintained at 37℃in a 5% (v/v) CO_2_ atmosphere and subcultured every 3 days. Transfection with siRNA against the HDAC3 gene (siHDAC3: 5’

-CCGCCAGACAAUCUUUGAAdTdT-3’) was performed using Lipofectamine® 2000. Scrambled siRNA was used as a control. Before transfection, the cells were synchronized with 100 ng/ml nocodazole for 16 h. Cell viability was determined on the indicated days using the Cell Counting Kit-8 according to the manufacturer’s instructions. The cell cycle of HepG2 after siRNA transfection was determined by flow cytometry analysis.

**HCC xenograft model**

SMMC-7721 cells (2×10^6^) were injected subcutaneously into 4-week-old male nude mice. Mice were sacrificed 6 weeks after injection and tumors were collected and examined. The tumor volume was measured using the following formula: length×width^2^×0.4. Three weeks after injection, mice were administered with panobinostat (5.0 mg/kg) or PBS intraperitoneally daily.^5^ Liver cancer cell lines SMMC-7721 and HepG2 were purchased from Cell Bank of Type Culture Collection of Chinese Academy of Sciences.

**SUPPLEMENTAL REFERENCES**

1. Hu W, Nevzorova YA, Haas U, Moro N, Sicinski P, Geng Y*, et al.* Concurrent deletion of cyclin E1 and cyclin-dependent kinase 2 in hepatocytes inhibits DNA replication and liver regeneration in mice. *Hepatology* 2014, **59**(2)**:** 651-660.

2. Duncan AW, Taylor Mh Fau - Hickey RD, Hickey Rd Fau - Hanlon Newell AE, Hanlon Newell Ae Fau - Lenzi ML, Lenzi Ml Fau - Olson SB, Olson Sb Fau - Finegold MJ*, et al.* The ploidy conveyor of mature hepatocytes as a source of genetic variation. *Nature* 2010(1476-4687).

3. Lee D, Xu IM-J, Chiu DK-C, Lai RK-H, Tse AP-W, Li LL*, et al.* Folate cycle enzyme MTHFD1L confers metabolic advantages in hepatocellular carcinoma. *The Journal of Clinical Investigation* 2017, **127**(5)**:** 1856-1872.

4. Wuestefeld T, Klein C, Streetz KL, Betz U, Lauber J, Buer J*, et al.* Interleukin-6/glycoprotein 130-dependent pathways are protective during liver regeneration. *Journal of Biological Chemistry* 2003, **278**(13)**:** 11281-11288.

5. Lachenmayer A, Toffanin S, Cabellos L, Alsinet C, Hoshida Y, Villanueva A*, et al.* Combination therapy for hepatocellular carcinoma: additive preclinical efficacy of the HDAC inhibitor panobinostat with sorafenib. *Journal of Hepatology* 2012, **56**(6)**:** 1343-1350.
