# Supplementary figures and images for: Histone deacetylase 3 promotes liver regeneration and liver cancer cells proliferation through signal transducer and activator of transcription 3 signaling pathway
Source: Cell Death Dis. 2018 Mar 14;9(3):398. doi: 10.1038/s41419-018-0428-x (PMC5852132; doi:10.1038/s41419-018-0428-x)

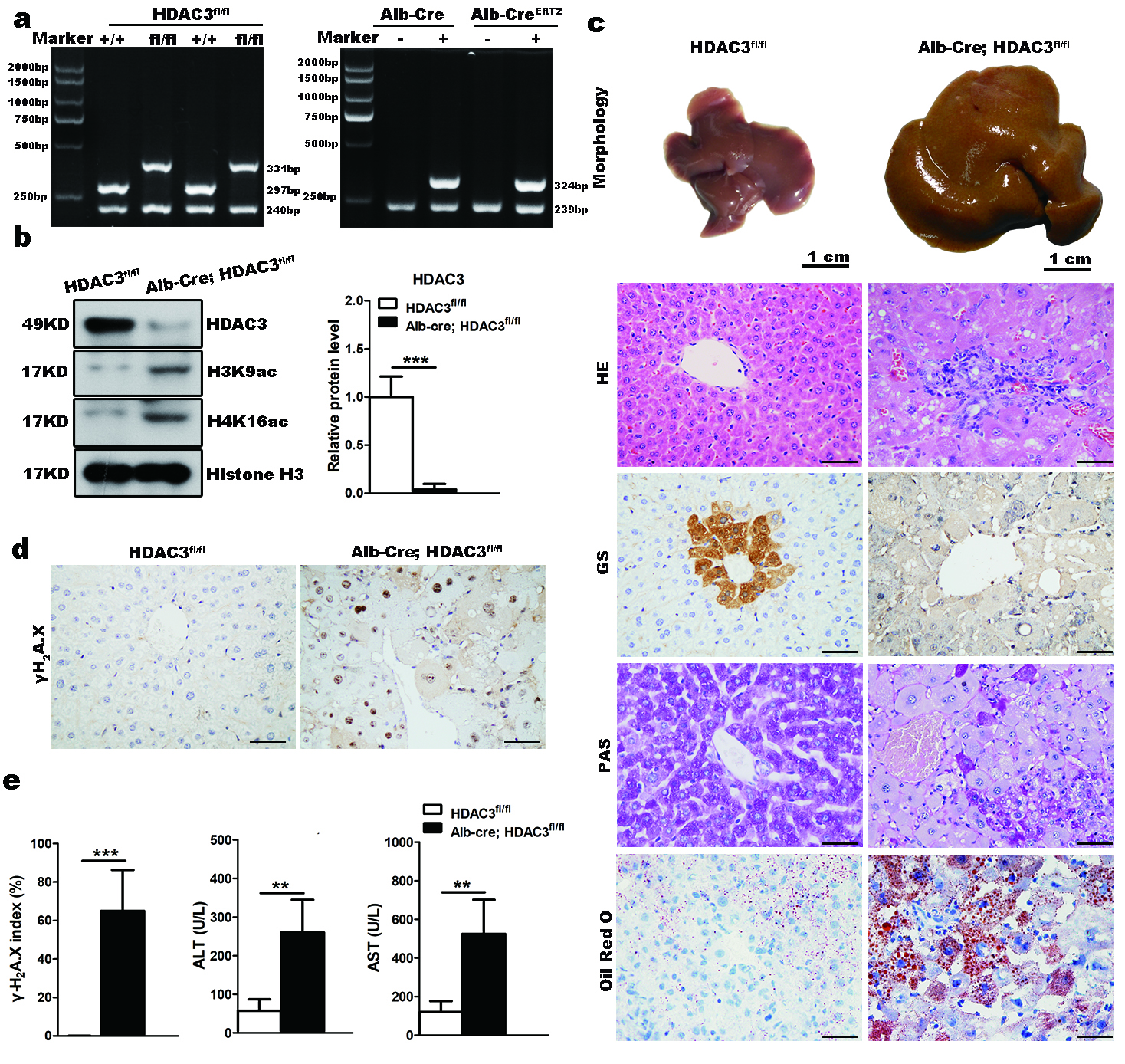

Supplement: Supplementary file 2 — Supplementary Figure1(JPG 2635 kb) [file 41419_2018_428_MOESM2_ESM.jpg]

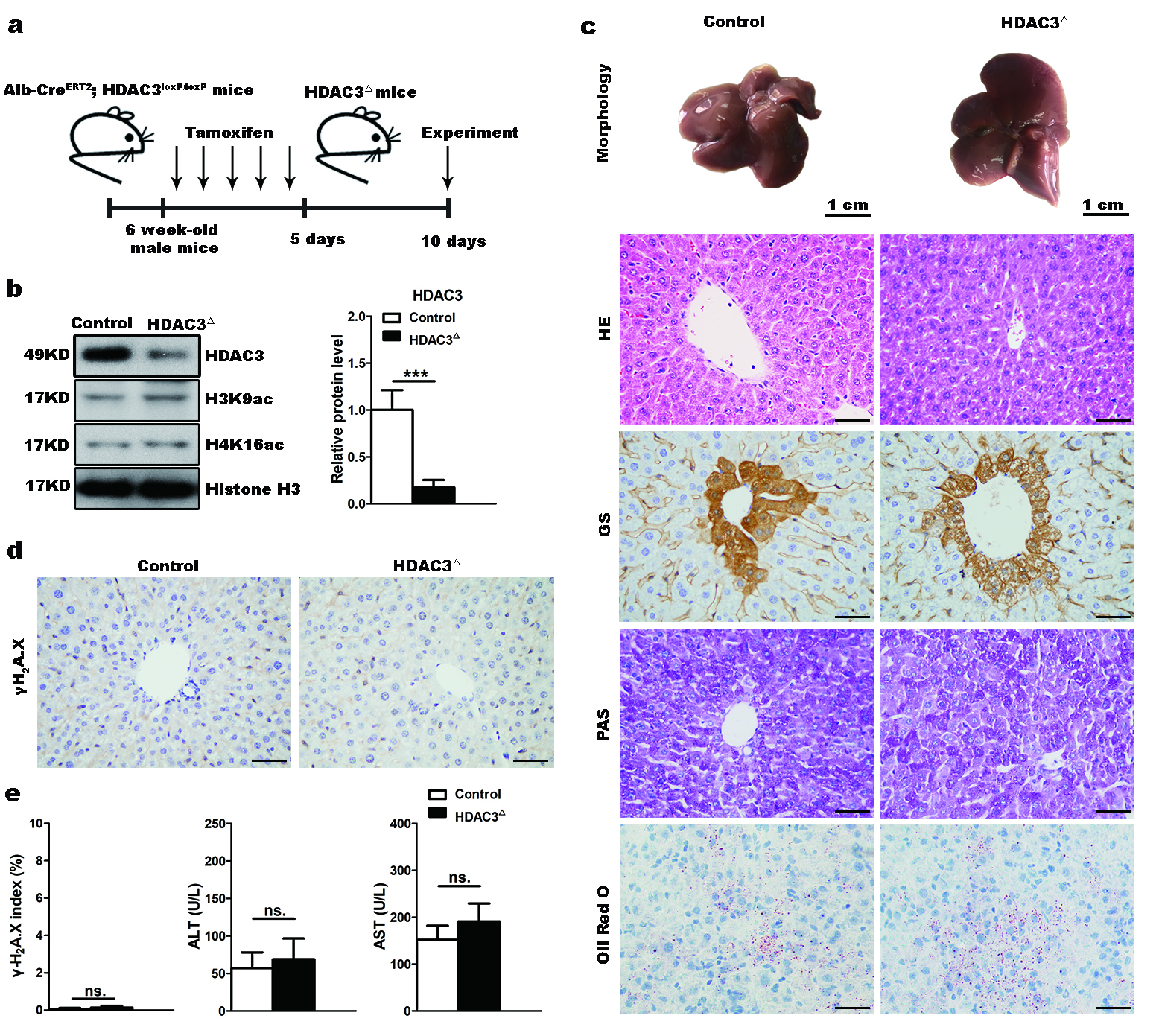

Supplement: Supplementary file 3 — Supplementary Figure2(JPG 2619 kb) [file 41419_2018_428_MOESM3_ESM.jpg]

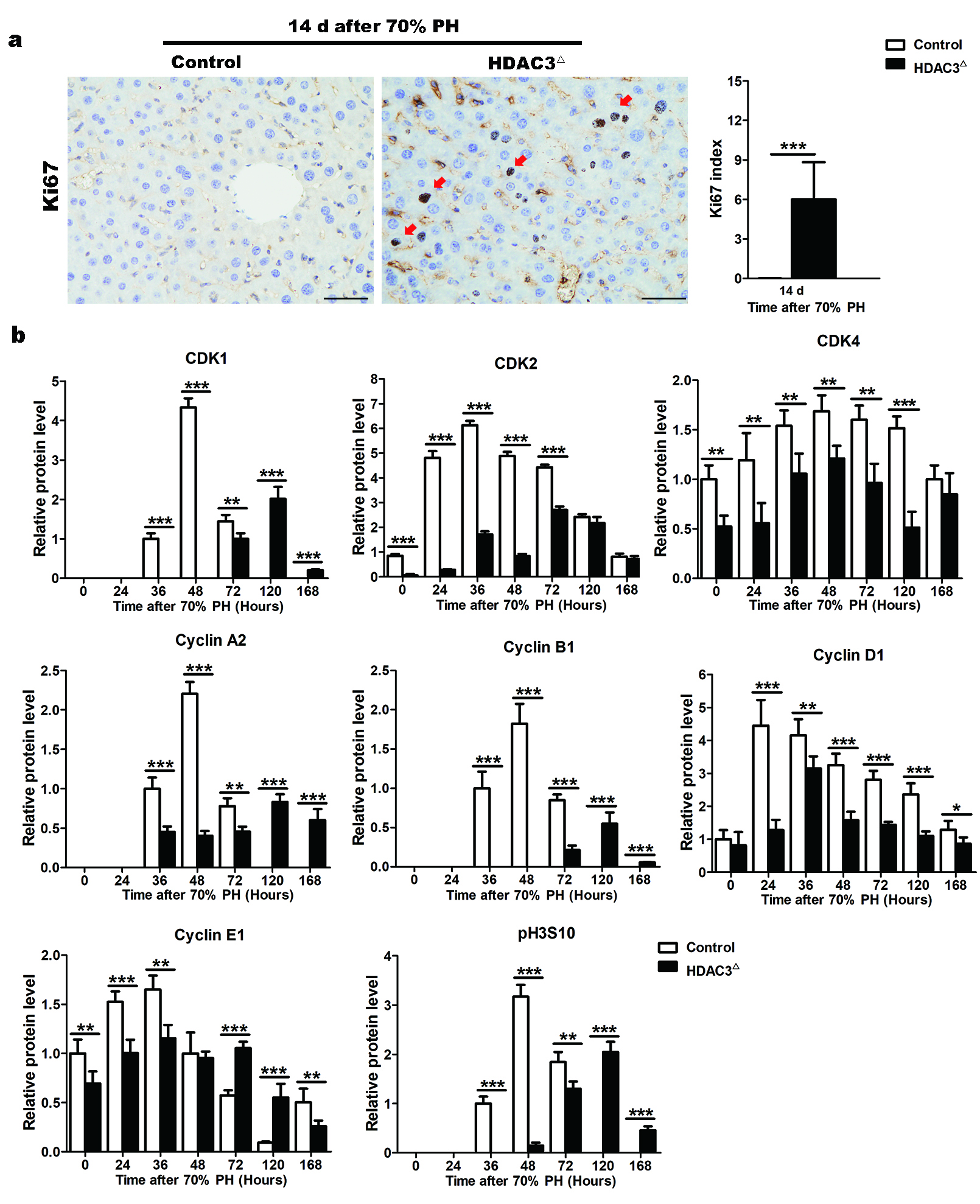

Supplement: Supplementary file 4 — Supplementary Figure3(JPG 2374 kb) [file 41419_2018_428_MOESM4_ESM.jpg]

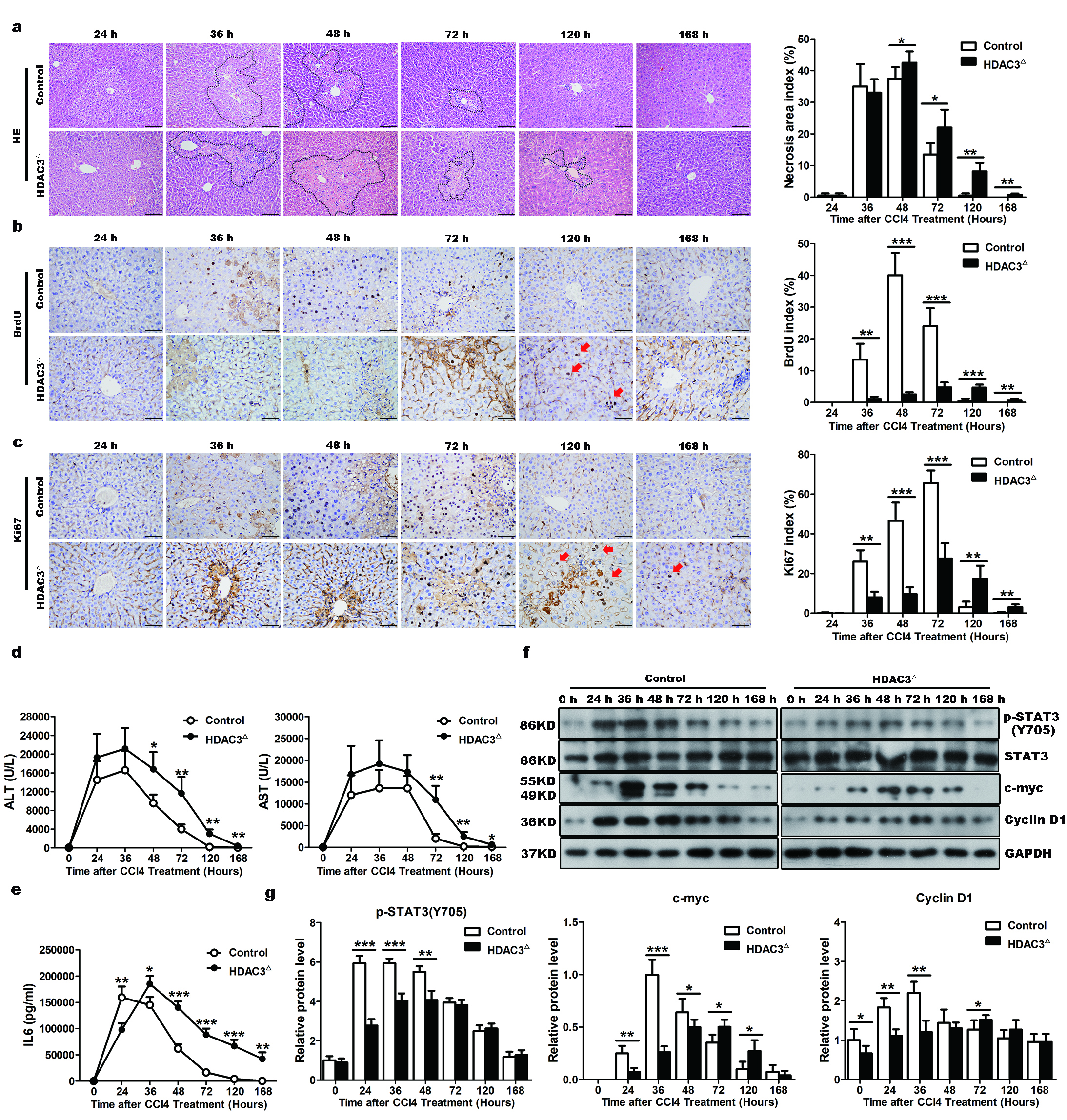

Supplement: Supplementary file 5 — Supplementary Figure4(JPG 5649 kb) [file 41419_2018_428_MOESM5_ESM.jpg]

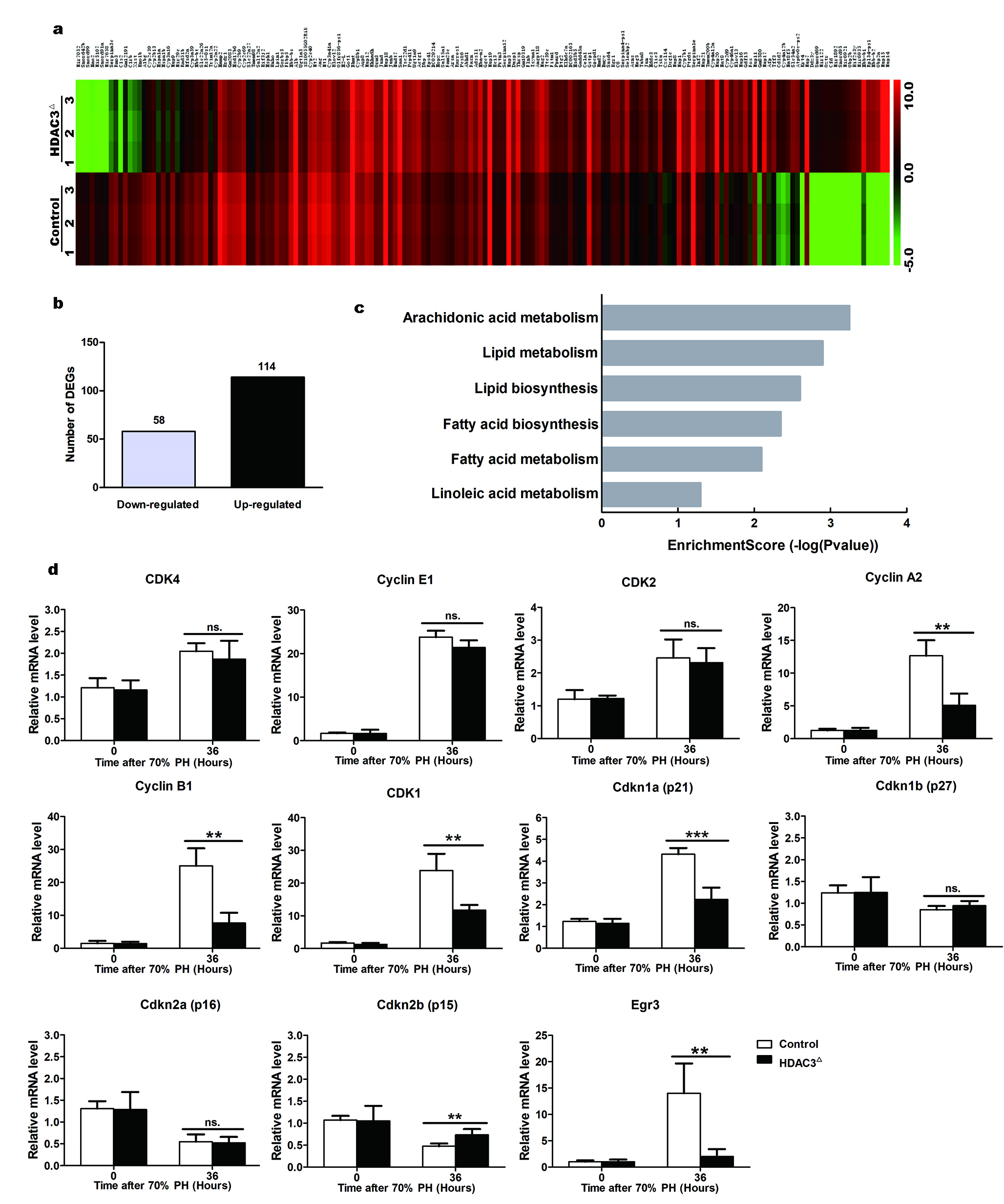

Supplement: Supplementary file 6 — Supplementary Figure5(JPG 2727 kb) [file 41419_2018_428_MOESM6_ESM.jpg]

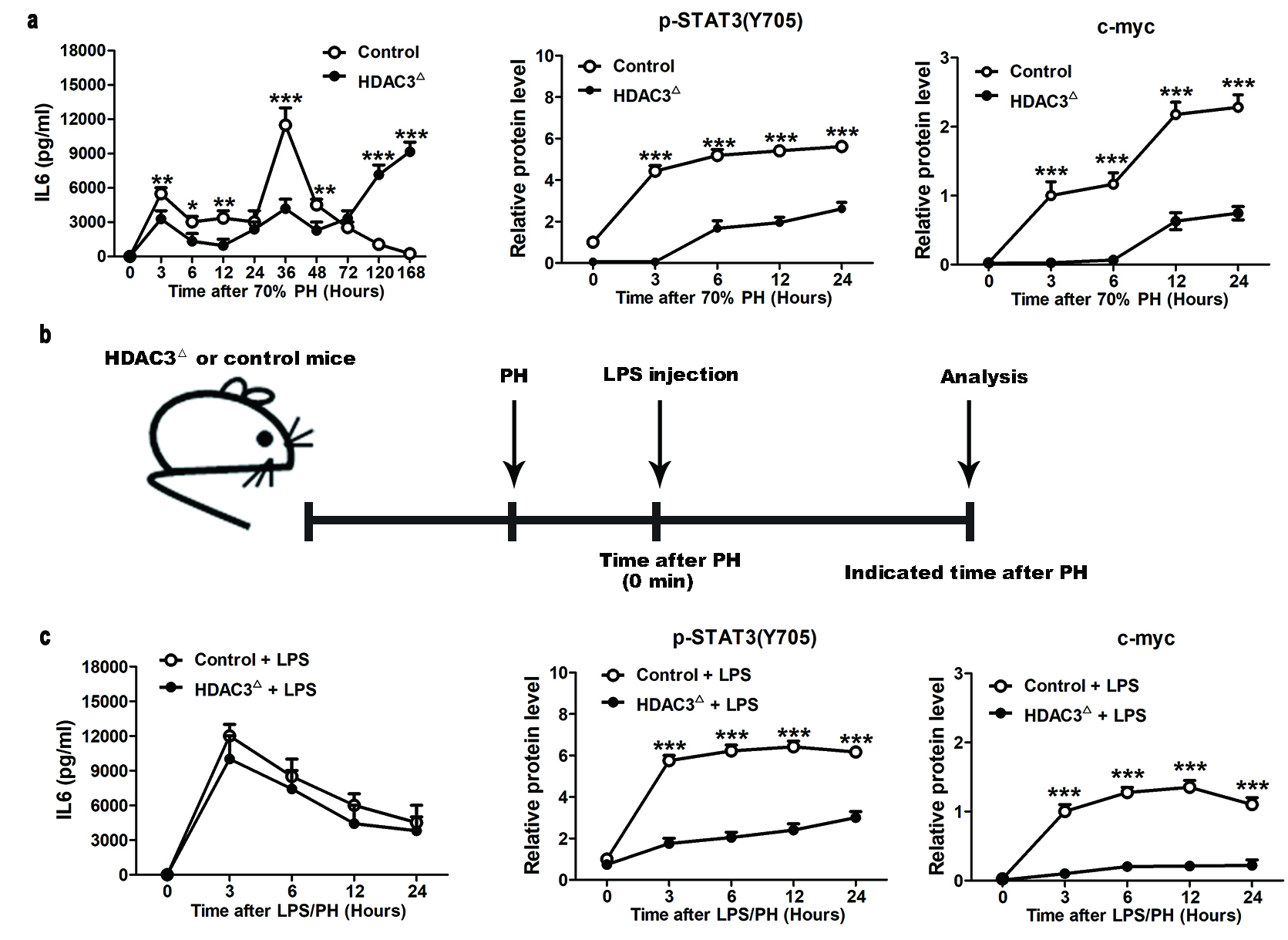

Supplement: Supplementary file 7 — Supplementary Figure6(JPG 1569 kb) [file 41419_2018_428_MOESM7_ESM.jpg]

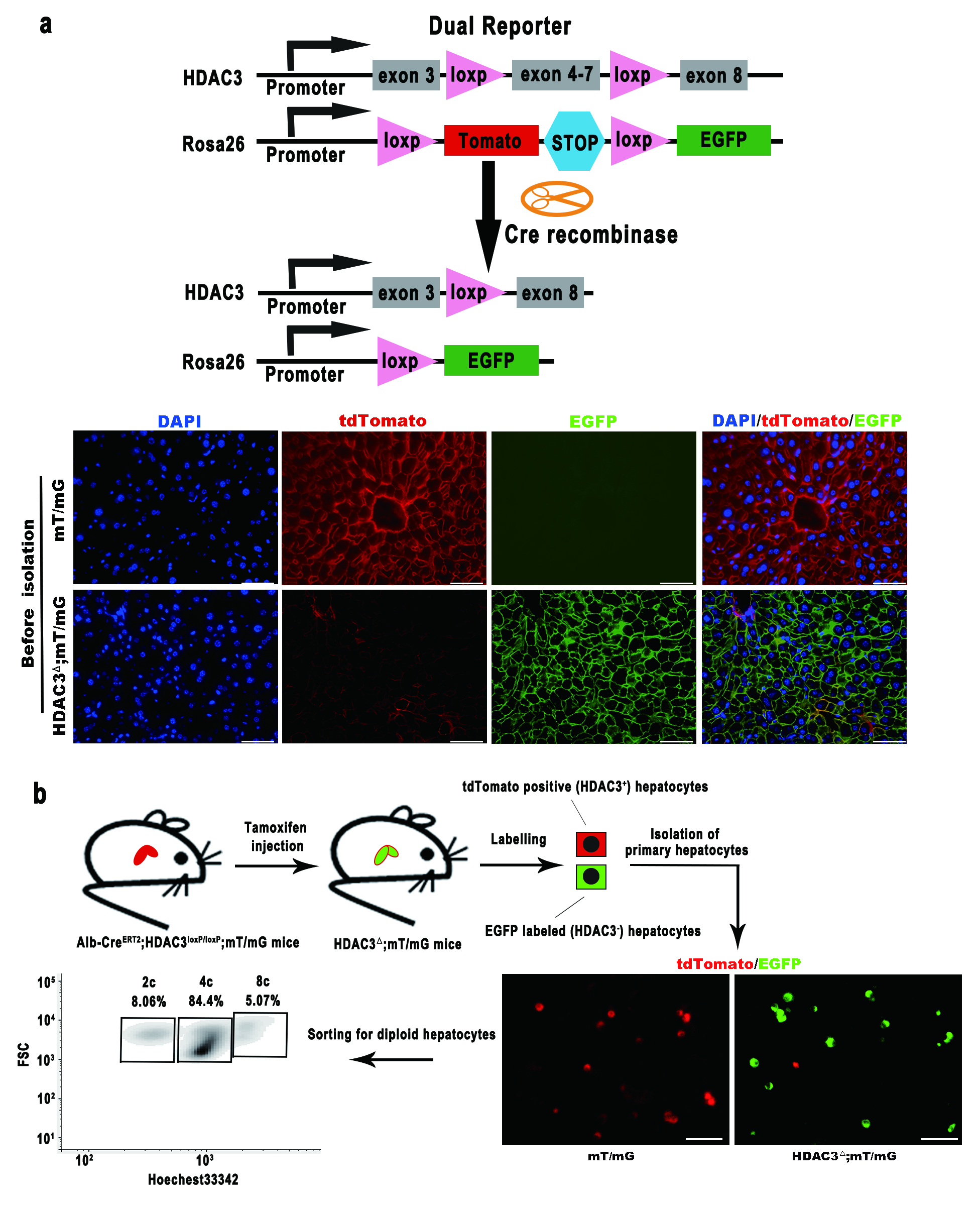

Supplement: Supplementary file 8 — Supplementary Figure7(JPG 3396 kb) [file 41419_2018_428_MOESM8_ESM.jpg]

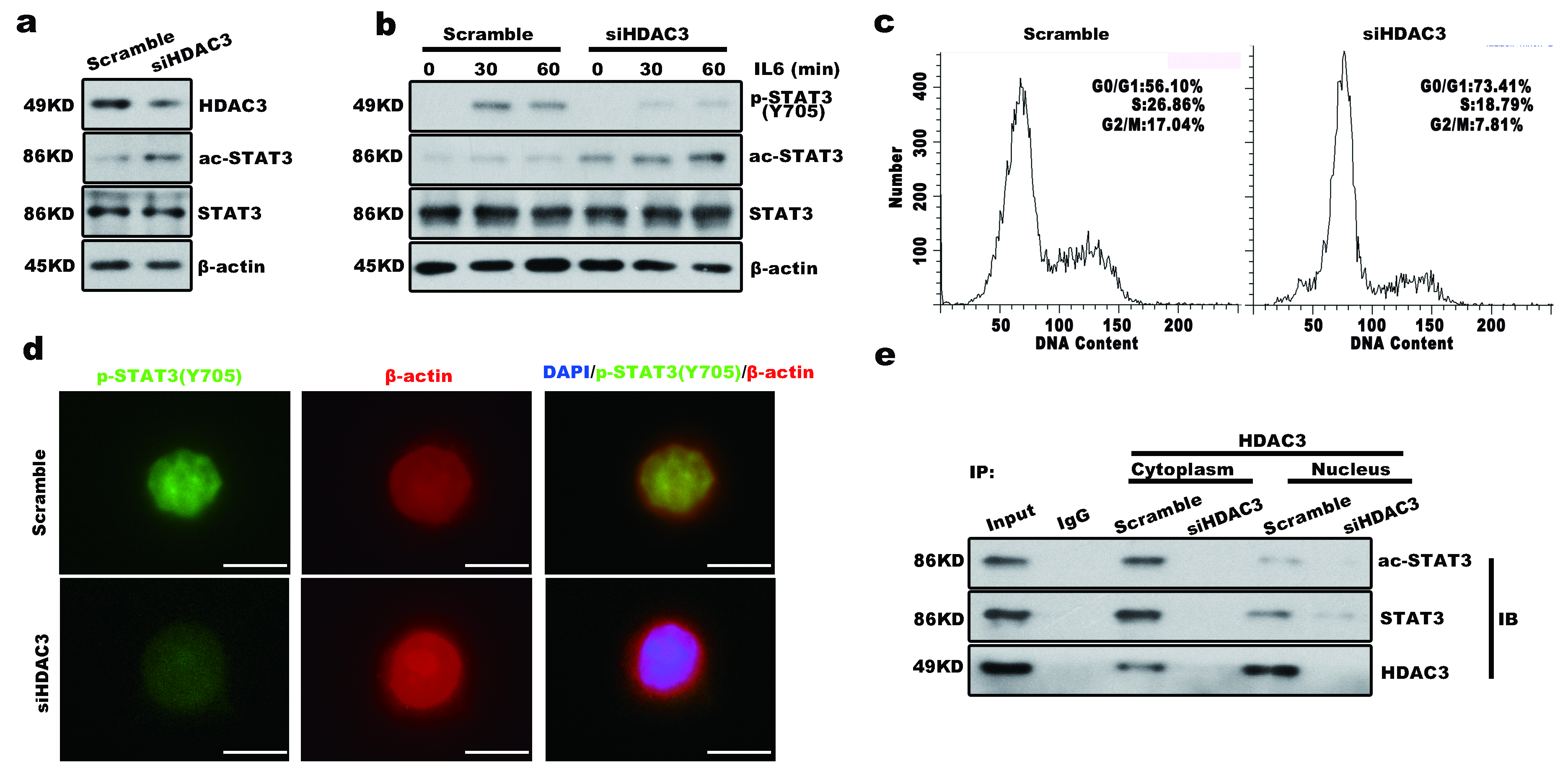

Supplement: Supplementary file 9 — Supplementary Figure8(JPG 4006 kb) [file 41419_2018_428_MOESM9_ESM.jpg]

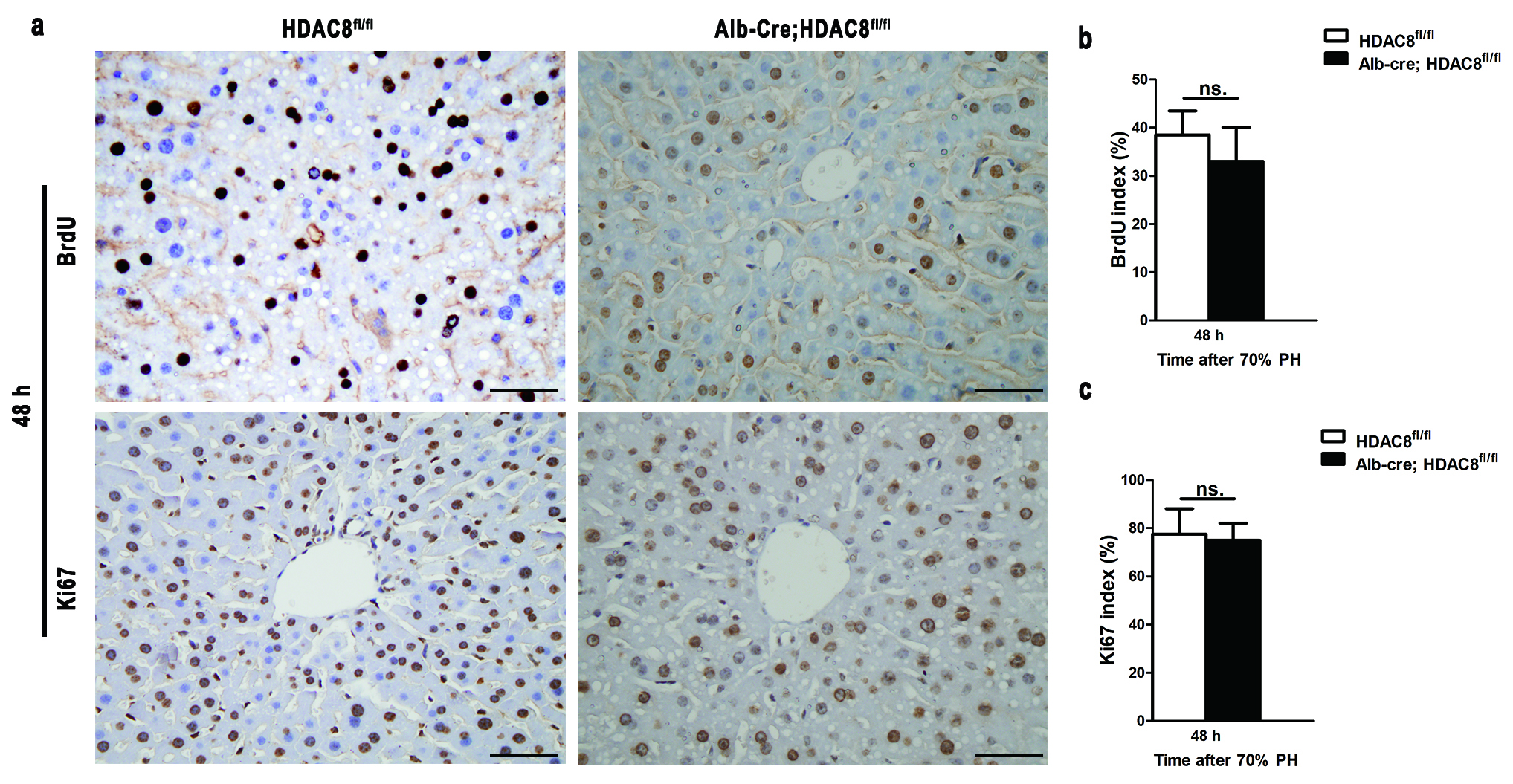

Supplement: Supplementary file 10 — Supplementary Figure9(JPG 2420 kb) [file 41419_2018_428_MOESM10_ESM.jpg]

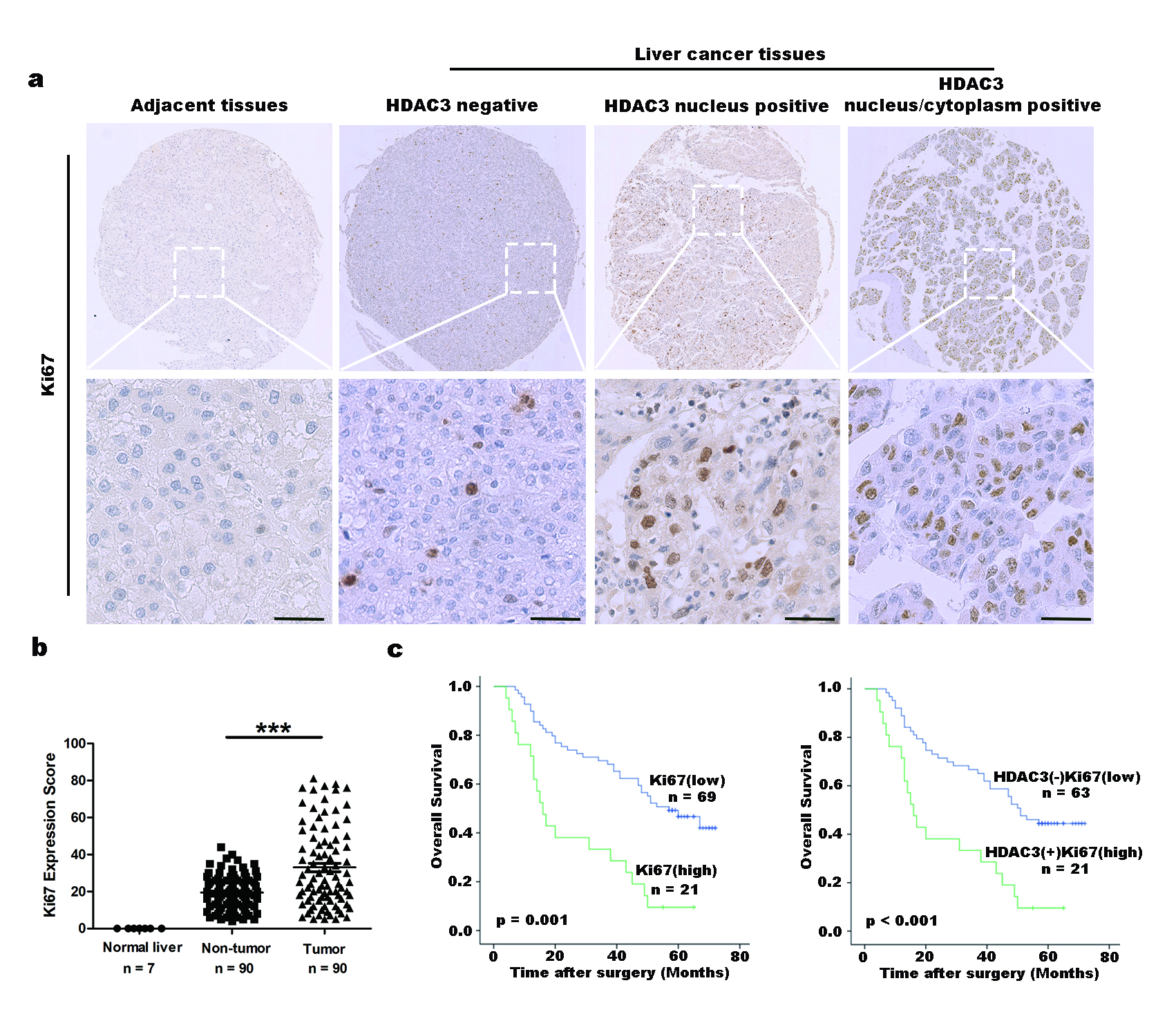

Supplement: Supplementary file 11 — Supplementary Figure10(JPG 2481 kb) [file 41419_2018_428_MOESM11_ESM.jpg]
